# Selective and rapid extraction of trace amount of gold from complex liquids with silver(I)-organic frameworks

**DOI:** 10.1038/s41467-022-35467-z

**Published:** 2022-12-15

**Authors:** Jie Luo, Xiao Luo, Mo Xie, Hao-Zhen Li, Haiyan Duan, Hou-Gan Zhou, Rong-Jia Wei, Guo-Hong Ning, Dan Li

**Affiliations:** College of Chemistry and Materials Science, Guangdong Provincial Key Laboratory of Functional Supramolecular Coordination Materials and Applications, Jinan University, Guangzhou, 510632 China

**Keywords:** Coordination chemistry, Environmental chemistry, Metal-organic frameworks

## Abstract

The design of adsorbents for rapid, selective extraction of ultra-trace amounts of gold from complex liquids is desirable from both an environmental and economical point of view. However, the development of such materials remains challenging. Herein, we report the fabrication of two vinylene-linked two-dimensional silver(I)-organic frameworks prepared via Knoevenagel condensation. This material enables selective sensing of gold with a low limit of detection of 60 ppb, as well as selective uptake of ultra-trace gold from complex aqueous mixtures including distilled water with 15 competing metal ions, leaching solution of electronic waste (e-waste), wastewater, and seawater. The present adsorbent delivers a gold adsorption capacity of 954 mg g^−1^, excellent selectivity and reusability, and can rapidly and selectively extract ultra-trace gold from seawater down to ~20 ppb (94% removal in 10 minutes). In addition, the purity of recovered gold from e-waste reaches 23.8 Karat (99.17% pure).

## Abstract (web summary)

The development of adsorbents for extracting gold from solutions is desirable from an environmental and economical point of view. Here, the authors report two two-dimensional silver(I)‒organic frameworks that enable selective sensing and extraction of gold from aqueous solutions, including seawater and solutions of e-waste.

## Introduction

Precious metals such as gold (Au) as indispensable resource have high value and widely used not only in catalysis^1,2^, bacteria resistance^3,4^, and anti-cancer drugs^5^ but also as curial materials for the electronics and jewelry industry^6,7^. The inappropriate disposal and dramatical boost of electronic waste (e-waste) have drawn massive environmental concerns due to the toxicity of gold salts in wastewater^8,9^. Moreover, the scarcity, huge consumption and increase demand lead to a continuously soaring gold price. It has been reported that approximately 300 tons of gold are used annually for manufacturing electronic products, and the gold concentrations reach 200–350 g per ton in electronic devices (e.g., mobile phone handsets and computer circuit boards), which are much richer than that in currently available ores^10,11^. Besides of e-waste, large quantities of gold are found in other secondary sources including incinerated sewage ash, wastewater, freshwater, and ocean^12–15^. Specifically, there are 20 million tons of gold in the ocean, with worth of 700 trillion US dollars^14,15^. However, the gold concentrations in the above-mentioned sources are extremely low, for example, less than 10 μg L^−1^ and 10 ng L^−1^ in wastewater and seawater, respectively^13^. In addition, these sources are usually complex liquids containing various kinds of organic compounds as well as competitive metals including sodium, potassium, copper, nickel, iron and rare earth metals. Some of the metals have a much higher concentration than gold, resulting in the difficulty in extraction processes. Therefore, the innovation of novel adsorbents that can rapidly, selectively concentrate the ultra-trace amount of gold at ppb or even at ppt level, is essential even though highly challenging.

Metal-organic frameworks (MOFs) are a class of crystalline porous materials that have attracted extensive attention due to their diverse structures, high surface area, chemical tunability and selective guest adsorptions^16–20^. Although a few MOFs have been demonstrated with the capability of gold adsorption, to our best of knowledge, MOFs that can concentrate ultra-trace gold (<20 ppb) from complex liquid have rarely reported so far (See Supplementary Table 12)^21–27^. Recently, we found that a Ag(I) cyclic trinuclear unit (CTU) can be used for rapid and selective sensing of Au(III) ion with a low limit of detection (LOD) of ~ 650 ppb via strong metallophilic attractions^28^. We hypothesize that the incorporation of Ag(I)-CTU into MOFs may be a promising strategy for improving the affinity of MOFs toward gold.

**Fig. 1:**
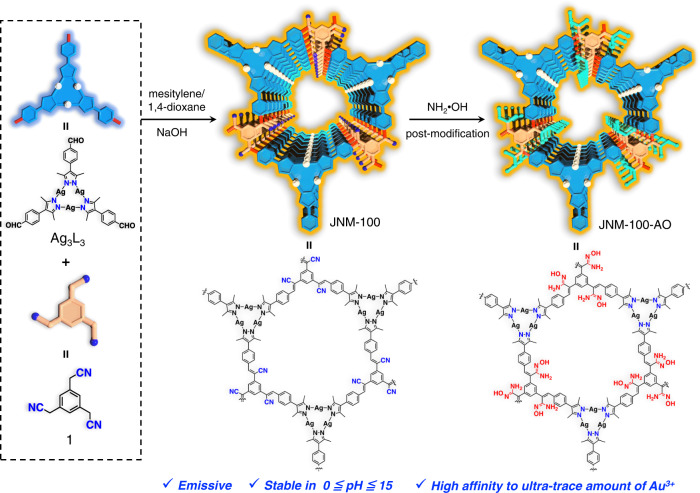
Fabrication and chemical structures of JNM-100 and JNM-100-AO. Left: The schematic representation and chemical structure of Ag_3_L_3_ and organic linker 1. Right: the schematic illustration and chemical structure of JNM-100 and JNM-100-AO.

In this work, by the integrating MOF and covalent organic framework (COF) chemistry^29,30^, we synthesize two highly stable, emissive and vinylene-linked two-dimensional (2D) Ag(I) CTU-based MOFs denoted as JNM-100 and JNM-100-AO (JNM stands for Jinan material) (Fig. 1). Due to the C=C bond linkages and fully conjugated structures, the JNMs feature good stability in acid/base in a pH ranging from 0 to 15, and good photoluminescent (PL) properties. JNM-100-AO shows excellent selectivity, reusability, and high loading capacity of 954 mg gold per g of absorbent, exceeding most of the reported MOFs (See Supplementary Table 12). More importantly, JNM-100-AO is able to selectively extract trace and ultra-trace gold from complex liquids including leaching solution of e-waste, distilled water, wastewater, seawater, with ultra-fast removal rate. We also demonstrate that JNM-100-AO can effectively extract gold with purities of 23.8 Karat from e-waste.

## Results and discussion

### Synthesis and characterization

**Fig. 2:**
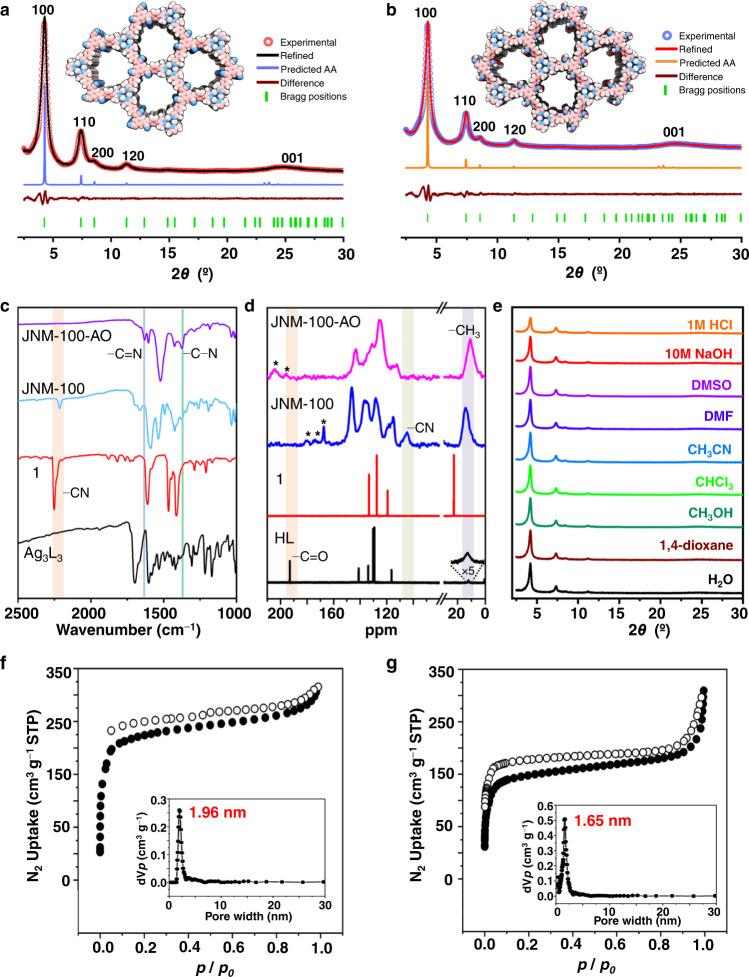
Characterization of JNM-100 and JNM-100-AO. Crystal structure of **a** JNM-100 and **b** JNM-100-AO showing space-filling model (C, light pink; H, white; Ag, silver; N, sky blue; O, red). **c** FT-IR spectra of JNM-100-AO, JNM-100, 1, and Ag_3_L_3_. **d** Solid-state ^13^C CP-MAS NMR spectra (100 MHz, 300 K) of JNM-100-AO, and JNM-100, and ^13^C NMR (100 MHz, 300 K, DMSO) spectra of linker 1 (red) and ligand HL (black) (*marked resonances represent spinning sideband). **e** The stability test of JNM-100-AO in strong acid and base as well as in various solvents. The N_2_ adsorption (filled) and desorption (open) isotherm profiles of **f** JNM-100, and **g** JNM-100-AO at 77 K. (inset the pore-size distribution curves of JNM-100-AO, and JNM-100 showing a uniform pore size of ∼1.96 and 1.65 nm, respectively).

The Ag_3_L_3_ (HL = 4-(3,5-dimethyl-1H-pyrazol-4-yl)benzaldehyde) were prepared according to previously reported methods (See the SI for details), and the reaction of Ag_3_L_3_ crystals, and organic linker (2,2′,2″-(benzene-1,3,5-triyl) triacetonitrile, 1) in a 5∶5∶1 (v/v) mixture of mesitylene, 1,4-dioxane, and 4 M aqueous NaOH led to green crystalline products JNM-100 (Fig. 1 and see the “Method”). The immersion of JNM-100 in an ethanol solution of NH_2_OH·HCl and Et_3_N at 85 °C for 24 h quantitatively gave brown-green crystalline products JNM-100-AO (Fig. 1). To understand the crystal structure of JNMs, powder X-ray diffraction (PXRD) experiments and theoretical simulations were conducted. As shown in Fig. 2a, the PXRD pattern of JNM-100 displayed an intense peak at 4.28° assigned to (100) diffraction, indicating the high crystallinity of JNM-100 with a long-range ordering in frameworks. In addition, peaks at 7.40°, 8.50°, 11.36°, and 24.94° correspond to (110), (200), (120), and (001) reflections, respectively, in good agreement with the simulated AA-stacking model. Pawley refinement was performed with the experimental PXRD data to give a hexagonal space group *P3* with unit cell parameters of *a* = *b* = 23.9030 Å, *c* = 3.8141 Å, with refinement parameters of *R*_p_ = 3.83% and *R*_wp_ = 4.13%. The refined PXRD patterns match well with the experimental data, as confirmed by the negligible difference plot in Fig. 2a. JNM-100-AO exhibited a similar AA-stacking structure with JNM-100 (See the SI and Fig. 2b), suggesting the amidoximation did not alter the structure and crystallinity.

The chemical structure of JNMs was confirmed through FT-IR, and solid-state ^13^C CP/MAS NMR analyses. Specifically, the significant reduction of C=O stretching signals at 1690 cm^−1^ corresponds to aldehyde group in the monomer and the appearance of a new characteristic band at 1660 cm^−1^ evidenced the formation of the C=C bonds in the as-prepared JNM-100 skeletons (Fig. 2c and Supplementary Fig. 2). In addition, the vanish of the C≡N stretching peak at 2235 cm^−1^ and the appearance of a new C=N stretching signal at 1630 cm^−1^ confirmed the conversion of C≡N groups to amidoxime groups in JNM-100-AO (Fig. 2c)^31,32^. Furthermore, the solid-state ^13^C CP/MAS NMR spectra of JNM-100 revealed that the C=O signals disappeared and the characteristic resonance peaks of −C≡N carbons at 105 ppm, further supporting the formation of C=C bonds. Compared to JNM-100, the resonance signals of −C≡N carbons at 105 ppm in ^13^C NMR of JNM-100-AO were completely evanesced, implying the successful and quantitative conversion form −CN to amidoxime group (Fig. 2c, d)^33^. JNMs both illustrated stalactite-shaped morphologies and consisted of highly crystalline lamellar structures (Supplementary Figs. 4 and 5) revealed by the SEM and TEM images. For instance, the TEM images of JNM-100 and JNM-100-AO both show a well ordered straight white-and-black stripes with a distance of 0.94 and 0.92 nm (Supplementary Figs. 6 and 7), respectively, corresponding to the projections of the (200) reflection plane, further supporting the eclipsed AA packing configuration. In addition, Energy-dispersive X-ray spectroscopy (EDS) elemental analysis of JNM-100 and JNM-100-AO revealed a uniform distribution of C, N, and Ag in their powder particles (Supplementary Figs. 8 and 10).

The permanent porosity of JNMs was evaluated by N_2_ sorption measurement at 77 K, which further confirmed their eclipsed AA stacking structures. The nitrogen adsorption isotherms of JNM-100 and JNM-100-AO both show the Type II adsorption profiles suggesting the microporous structure (Fig. 2f, g). The Brunauer−Emmett−Teller (BET) surface areas of JNM-100 and JNM-100-AO were calculated to be 729 and 504 m^2^ g^−1^, respectively. The average pore size distribution of JNMs was found to be ∼1.96 and ~1.65 nm using the nonlocal density functional theory (NLDFT), consistent with theoretical values of 1.90 and 1.83 nm predicted for JNM-100 and JNM-100-AO, respectively (Fig. 2f and g). Thermal gravimetric analyses (TGA) and various temperature PXRD experiments under the N_2_ atmosphere are indicative of the moderate thermal stability of JNMs that their crystallinity remained up to 300 °C (Supplementary Figs. 12-14). Interestingly, JNMs exhibited good stability in various organic solvents, water, HCl and NaOH. It is worthy to emphasized that JNMs can maintain their crystallinity even after suspending in the aqueous solution of 10 M NaOH (pH = 15) and 1 M HCl (pH = 0) (Fig. 2e and Supplementary Figs. 15-16). Moreover, the surface areas of JNM-100 and JNM-100-AO exhibited slightly decline from 729 and 504 m^2^ g^−1^ to 613 and 493 m^2^ g^−1^, respectively, after treatment with 1 M HCl (Supplementary Fig. 18), further confirming their high stability under acidic condition.

### PL properties and detection of Au^3+^ ions

**Fig. 3:**
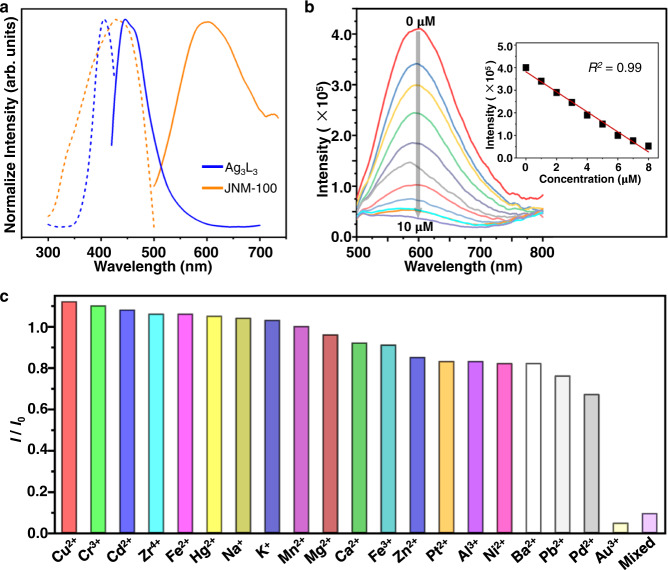
Photoluminescent (PL) and selective detection of gold ions of JNM-100. **a** Excitation emission spectra of Ag_3_L_3_ and JNM-100 (blue and orange present PL spectra of Ag_3_L_3_ and JNM-100, respectively; dashed and solid lines represent excitation and emission spectra, respectively). **b** Fluorescence spectra of JNM-100 in the presence of an increasing concentration of Au(III) (from 0 to 10 μM) in water and methanol mixture (50:50 v/v%). Inset, profiles of the emission intensity *versus* Au(III) concentration showing a good linear relationship. **c** Comparison of the relative emission intensity ratio (*I/I*_*0*_) indicates indicating a good selectivity for Au(III) detection over 20 metal ions for JNM-100.

JNMs displayed orange red PL either in the solid state or in solution (Fig. 3a and Supplementary Fig. 28). Upon excitation at 460 nm, JNM-100 and JNM-100-AO exhibited a maximum emission band (λ_em_) at 605 and 602 nm with an absolute QY of 4.1% and 3.4%, respectively. In contrast, the Ag_3_L_3_ monomer showed an emission band at 445 nm, the large red-shift (~160 nm) of emission of JNMs from that of the Ag_3_L_3_ monomer can be attributed to the extended π-conjugation and the interlayers metalophilic interaction (Fig. 3a and Supplementary Fig. 28). In addition, JNMs are highly emissive in various solvents including H_2_O, methanol, chloroform, and THF, expanding their application scope in as fluorescent sensing (Supplementary Figs. 27 and 29).

To test the performance of Au(III) detection, it was found that the PL intensity of JNM-100 gradually decreased with the increasing Au(III) concentration and over 99% PL intensity were quenched with the addition of 10 μM Au(III) (Fig. 3b). The profiles of PL intensity *versus* Au(III) concentration displayed a linear relationship in the range of 0–8 μM (Fig. 3b), and LOD is 60 ppb for JNM-100. The introduction of amidoxime on the pore surface did not affect the Au(III) sensing performance of JNM-100-AO and it exhibited a similar LOD of 66 ppb (Supplementary Fig. 31). More interestingly, JNMs can selectively detect Au^3+^ over 20 different metal ions. The influence of counter ions was firstly examined, and the fluorescence intensity of JNMs displayed negligible change by altering anions including Cl^–^, Br^–^, Ac^–^, NO_3_^–^, and SO_4_^2–^ (Supplementary Fig. 35). When JNMs were suspended in a methanol/water solution (v/v, 1:1) containing 10 ppm Au(III), the PL intensity of JNMs was rapidly and significantly quenched. In sharp contrast, other 20 kinds of metal ions such as Cu^2+^, Ni^2+^, Fe^2+^, Fe^3+^, Al^3+^, and so on, with 10 times higher concentration (*i.e*.,100 ppm) only slightly induced PL changes (Fig. 2c and Supplementary Fig. 34). Such PL quenching might be attributed to the strong metal-metal interactions between Au(III) and Ag(I)-CTUs in the frameworks, resulting in enhancement of radiative decay^28^. To further prove this hypothesis, we prepared a COF, TFPT-BTAN (see the SI)^33^ as a reference with similar pore size and nitrile groups but without Ag(I)-CTUs. Indeed, TFPT-BTAN did not show any noticeable PL quenching with 100 ppm gold, further confirming Ag(I)-CTUs play a curial role in interacting with Au(III) (Supplementary Fig. 32). Noticeably, although the JNMs delivered higher LODs for Au(III) in pure water (Supplementary Figs. 36 and 37) than those in the methanol/water mixed solvent, JNMs can be used to detect trace amount of gold ions in real industrial waste (i.e., gold plating waste water) (Supplementary Fig. 39 and see the SI for details).

### Gold adsorption performance

**Fig. 4:**
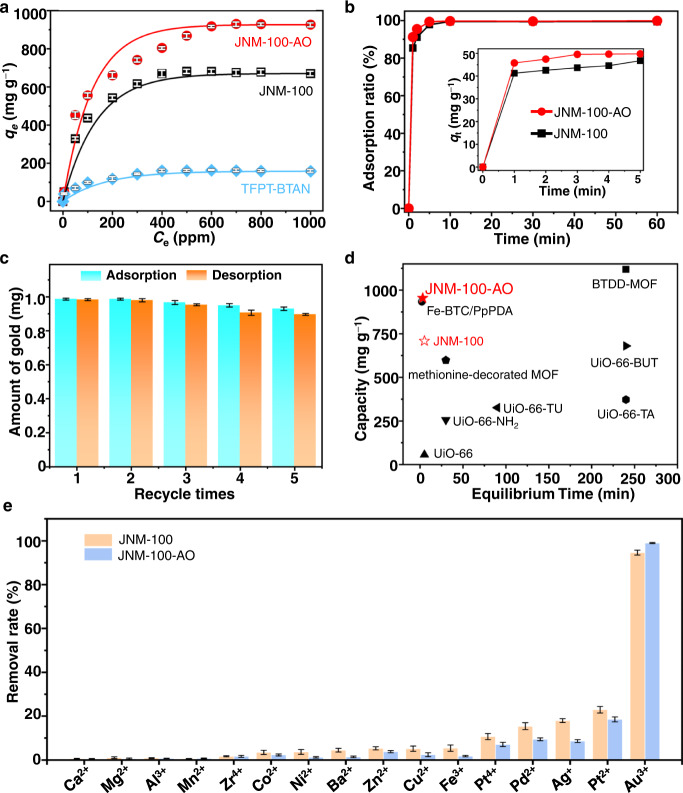
Selective gold adsorption and extraction using JNMs. **a** Adsorption isotherms of gold at the initial concentration range of 5–1000 ppm at rt using JNM-100-AO, JNM-100, and TFPT-BTAN, suggesting superior affinity of JNM-100-AO toward gold (*C*_e_ represents the equilibrium concentration and *q*_e_ represents the equilibrium adsorption capacity). **b** Kinetic studies of gold adsorption at an initial concentration of 50 mg L^−1^ for JNM-100 and JNM-100-AO, respectively (inset: expansion of the first 5 min, where *q*_t_ represents the adsorption capacity at a certain time). **c** Recyclability test of JNM-100-AO for adsorption (cyan) and extraction (orange) amount of gold (10 mg JNM-100-AO was added to 10 mL gold solution containing 1.0 mg of gold). **d** The evaluation of reported MOF adsorbents performance. **e** Comparison of removal rate of metals using JNM-100 (light orange) and JNM-100-AO (light blue), implying good selectivity of JNMs for Au^3+^ uptake (initial concentration of competitive metal ions is 50 ppm; the initial concentration of Au^3+^ is 1 ppm). The error bar in (**a**), (**c**), and (**e**) represents the standard deviation of three independent measurements.

The good selective recognition of Au(III) using JNMs promotes us to further investigate their ability for gold adsorption. Studies of the adsorption performance of JNMs at different pH values found that the optimal acidity for the adsorption of Au(III) was at pH = 4 and 2 for JNM-100 and JNM-100-AO (Supplementary Fig. 40), respectively. Temperature effects have also been considered, and the adsorption of gold were gradually decreased when temperature increased (Supplementary Fig. 42). With these results, the adsorption isotherms of gold were recorded at room temperature (rt) and pH = 2, the conditions that JNMs would remain high crystallinity and structural integrity. Typically, JNMs (10 mg) was added into 10 mL Au(III) aqueous solution with different concentrations (*C*_0_ = 0–1000 ppm), and the results revealed that the adsorption capacity of JNMs enhanced until the equilibrium was reached with the increase of gold concentration (Fig. 4a). In addition, the adsorption isotherms of JNMs were fitted with the Langmuir isotherm model with a correlation coefficient >0.99, implying the Au(III) were mainly adsorbed as monolayer in JNMs. The Langmuir constant *K*_L_ and the maximum adsorption capacity (*q*_m_) of JNM-100-AO were calculated to be 0.052 L mg^–1^ and 954 mg g^–1^, respectively (See Method and Supplementary Table 7). For comparison, the reference COF TFPT-BTAN only showed a much lower *q*_m_ of ~167 mg g^−1^ (Fig. 4a and Supplementary Table 7), further indicating the introduction of Ag(I)-CTUs can readily enhance the adsorption capacity. More interestingly, the JNM-100-AO can deliver *q*_m_ of 767 mg g^–1^ at very low gold concentration of 1 ppm (Supplementary Fig. 45), which is rarely achieved by other adsorbents. Furthermore, JNMs exhibit fast kinetics for gold adsorption and reach the >99% removal rate within 5 and 3 mins for JNM-100 and JNM-100-AO, respectively (Fig. 4b). JNM-100-AO has much higher adsorption capacity and much faster kinetics than most reported MOFs (Fig. 4d and Supplementary Table 12)^21–27^.

The selective uptake of gold in complex systems containing potential competition metals is crucial for gold recovery. The addition of JNM-100-AO into a solution containing 50 ppm Ca^2+^, Mg^2+^, Al^3+^, Mn^2+^, Zr^4+^, Co^2+^, Ni^2+^, Ba^2+^, Zn^2+^, Cu^2+^, and Fe^3+^, respectively, only 0.8–9.1% metal ions were adsorbed, whereas 99.1% Au^3+^ (1 ppm) can be removed (Fig. 4e). It is known that [PtCl_4_]^2−^ has similar planar conformation, and ion radius with [AuCl_4_]^−^. The distinction between Au^3+^ from Pt^2+^ is hard to be achieved^34–38^. Surprisingly, JNM-100-AO exhibits higher affinity toward Au^3+^ compared to Pt^2+^. 99.1% of Au^3+^ (*C*_0_ = 1 ppm) can be absorbed, while only 18.5% of Pt^2+^ (*C*_0_ = 50 ppm) was removed (Fig. 3e). The relevant simulated distribution coefficient (*k*_d_) of Au^3+^ and Pt^2+^ are calculated to be 1.1×10^5^ and 227 mL g^−1^, respectively, revealing that JNM-100-AO is ~487 times more selective for Au^3+^ than Pt^2+^. To further elucidate the origin of this selectivity, the density functional theory (DFT) calculations were conducted to understand the interactions between [PtCl_4_]^2−^ or [AuCl_4_]^−^ and JNMs. The corresponding binding energy (*E*_b_) between [AuCl_4_]^−^ and Ag(I)-CTUs in JNM-100 was calculated to be −0.478 eV, and a short distance of 3.222 Å between Ag^+^ and Au^3+^ ions was observed, indicating strong attractive interactions may occur. In contrast, the *E*_b_ between [PtCl_4_]^2−^and Ag(I)-CTUs in JNM-100 was −0.268 eV, and a distance of 3.277 Å between Ag^+^ and Pt^2+^ ions was founded, suggesting much weaker interactions (See the SI). Besides the selectivity, the efficient extraction of Au^3+^ ions from JNMs and the stability of JNMs are essential to the recyclability for practical application. The adsorbed Au^3+^ ions can be easily desorbed by being treated with thiourea solution, giving rise that 0.98 mg of Au^3+^ ions (total adsorbed gold is 0.99 mg) were removed from JNM-100-AO in the first cycle. Even after five absorption-desorbed cycles, regenerated JNM-100-AO can still absorb 0.92 mg of Au^3+^ (total 1.0 mg gold in solution), and 0.89 mg of Au^3+^ can be desorbed with thiourea (Fig. 4c). More importantly, the crystallinity of JNM-100-AO remains unchanged (Supplementary Fig. 46), and the surface area of JNM-100-AO only show a slightly decrease (Supplementary Fig. 18) after five cycles. These results confirmed that JNM-100-AO is a promising adsorbent for gold recovery with excellent reusability.

### Mechanistic investigation

**Fig. 5:**
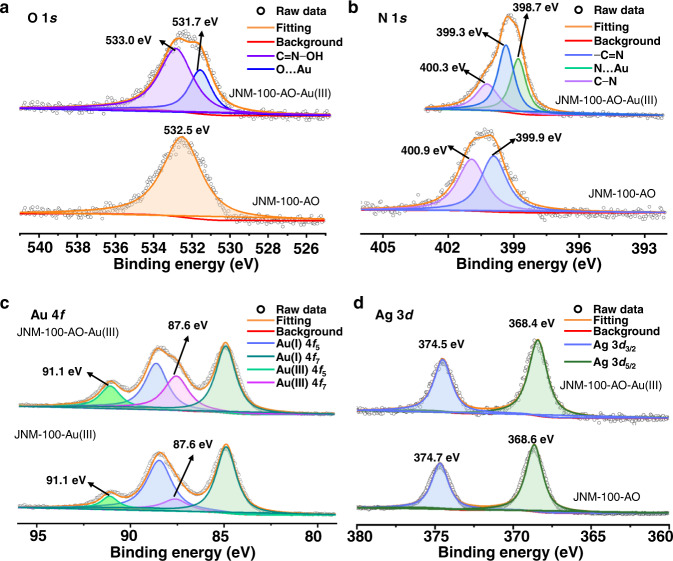
Mechanistic investigation with XPS analysis. XPS spectra of **a** O 1*s*, **b** N1*s*, and **d** Ag 3*d* for JNM-100-AO (before adsorption of Au^3+^) and JNM-100-AO-Au(III) (adsorbed Au^3+^ samples). **c** XPS spectra of Au 4 f for JNM-100-Au(III) and JNM-100-AO-Au(III).

To reveal the adsorption mechanism, thermodynamic analysis, X-ray photoelectron spectroscopy (XPS) and computational simulation were conducted. As shown in Supplementary Table 6, the values of Gibbs energy change (Δ*G*), enthalpy change (Δ*H*) and entropy change (Δ*S*) of JNMs are negative, indicating the adsorption of Au^3+^ ions on JNMs is a spontaneous process and enthalpy-favored (See the SI and Supplementary Table 6). The more negative Δ*G* of JNM-100-AO than that of JNM-100 suggests that JNM-100-AO is more energy favorable for adsorbing Au^3+^ ions. In addition, XPS spectra of JNMs before and after uptake of [AuCl_4_]^−^ were recorded to disclose the strong bonding site. Taking JNM-100-AO as an example, Fig. 5a shows only one peak attributed to the binding energy of O 1*s* for C=N−OH before the adsorption of gold, while a new peak appears at 531.7 eV might be assigned to the interaction between C=N−OH and [AuCl_4_]^−^ (Fig. 5a). In addition, XPS spectra of JNM-100-AO-Au(III) (absorbed gold sample), not only revealed the characteristic signals of N 1*s* for C=N and N−H with a binding energy of 399.7 and 400.6 eV, respectively, but also show a new peak at 398.9 eV, which could be assigned to the interactions between C=N and [AuCl_4_]^−^ (Fig. 5b). These results confirm that the introduction of amidoxime groups could bring new bonding sites for gold, resulting in the enhancement of adsorption capacities. Furthermore, compared to JNM-100-AO, the wide XPS spectra of JNM-100-AO-Au(III) clearly showed the Au 4 *f* peaks, supporting gold was indeed adsorbed (Supplementary Fig. 50). Such results are also consistent with the EDS analysis of JNM-100-AO-Au(III), which unambiguously shows the uniform distribution of element Au (Supplementary Fig. 11). Compared to the reported characteristic signals of Au^3+^ 4*f* at 87.9 and 91.5 eV^39^, JNM-100-AO-Au(III) displayed lower binding energy located at 87.6 and 91.1 eV (Fig. 5c). Meanwhile, JNM-100-AO exhibited the characteristic peaks of Ag 3*d* located at 368.7 and 374.7 eV, which were moved to lower binding energy of 368.4 and 374.5 eV after loading gold (Fig. 5d). Moreover, JNM-100-AO-Au(III) showed a peak attributed to Au−Cl at 200.2 eV remarkably higher position than the characteristic peak of Au−Cl in [AuCl_4_]^−^ (199.3 eV)^40^, suggesting strong interactions between Au−Cl and Ag(I) CTU (Supplementary Fig. 53). These experimental results evidenced that Ag(I)-CTUs in JNMs are beneficial for loading [AuCl_4_]^−^ ions.

Periodic DFT calculations within the CASTEP module in Materials Studio 2018 (See the Method and SI for details) were conducted to further search for the possible interaction sites of adsorption. Firstly, metallophilic interactions between *d*^10^ metal ions were widely observed. Thus, these interactions were considered to be the main active sites herein. The interaction mode that [AuCl_4_]^−^ lying above the JNM-100 layer was fully optimized. The corresponding binding energy (*E*_b_) between [AuCl_4_]^−^ and JNM-100 was calculated to be −0.478 eV and Van der Waals weak interactions were also observed (Supplementary Fig. 54), indicating the strong attractive interaction. As a comparison, the interaction mode between the TFPT-BTAN plane and [AuCl_4_]^−^ around the triazine ring at the same theoretical level was simulated. The periodic DFT calculation results showed a rather long Au−N distance (3.731 Å) (Supplementary Fig. 54). In addition, the *E*_b_ between [AuCl_4_]^−^ and triazine ring on TFPT-BTAN is calculated to be 0.187 eV, which is much smaller than that between [AuCl_4_]^–^ and Ag(I)-CTU on JNM-100. Secondly, another potential adsorption site is proposed to be around CN groups, in which metal-π interaction could stabilize the adsorption structure. The optimized adsorption structures in the CN site of JNM-100 and contrasted TFPT-BTAN are shown in Supplementary Figs. 54b and 54d. The *E*_b_ were calculated to be −0.092 eV and 0.237 eV, respectively, revealing that the CN group in JNM-100 showed a higher affinity toward [AuCl_4_]^–^. These theoretic results are in good agreement with the experimental observation and further proved that Ag(I) CTUs on JNMs play a curial role in the adsorption of gold.

### Extraction of ultra-trace amounts of gold

**Fig. 6:**
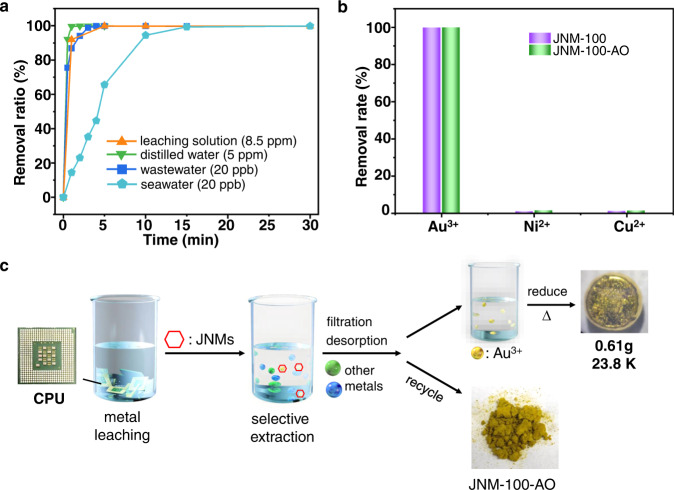
Gold recovery from complex liquids using JNM-100-AO. **a** The extraction rate of gold from complex liquids using JNM-100-AO. The complex liquids are e-waste leaching solution obtained from the CPU (*C*_0_ = 8.5 ppm), orange line; distilled water containing 5 ppm gold, green line; wastewater obtained from chicken (*C*_0_ = 20 ppb), blue line; and seawater obtained from the South China Sea (*C*_0_ = 20 ppb), cyan line. **b** Extraction of gold from a CPU showing the removal rate of the metals including Au^3+^, Ni^2+^ and Cu^2+^ ions with JNMs. **c** Practical application of JNM-100-AO for gold recovery from e-waste.

Since JNMs exhibited good adsorption capacity, rapid kinetics and excellent reusability for gold adsorption, the application for trace or ultra-trace amounts of gold extraction was also tested. The distilled water containing 5 ppm of gold was treated with JNM-100-AO. Over 92% of Au^3+^ is extracted from the water within 30 s. It reaches the equilibrium with removal efficiency of 99% after 1 min (Fig. 6a). A more complex system such as wastewater and seawater with much lower gold concentrations was further tested. JNM-100-AO (20 mg) was added to 5 L of wastewater, obtained from kitchen water, in which the concentration of gold was evaluated to be 20 ppb. Over 94% of Au^3+^ is extracted from the wastewater within 2 min, and under 3 min over 99% of Au^3+^ is removed, which is rarely achieved by other adsorbents (Fig. 6a). Finally, the ultra-trace amount of gold was added to real seawater obtained from the South China Sea (5 L) and the concentration of Au^3+^ was determined to be 20 ppb. The seawater solutions were then treated with 20 mg of JNM-100-AO. Remarkably, in 10 min, 95% of Au^3+^ is recovered and 99% of Au^3+^ is extracted within 15 min (Fig. 6a). These results indicated that JNM-100-AO exhibited an extraordinary ability to concentrate ultra-trace amounts of gold with high selectivity and compatibility, even against the high concentration of organic compounds, microorganisms and other competitive metals in wastewater and seawater. However, it is noteworthy that the concentration of gold in real seawater is much lower, and JNMs might be not suitable for extracting gold from oceans at this stage.

### Practical application of gold recovery from e-waste

The disposed computer processing units (CPUs) were pretreated according to reported procedures (See Method and SI for details)^41^. The resulting leaching solution contained a metal composition of Au^3+^, Ni^2+^ and Cu^2+^ with concentrations of 8.5, 468.2, and 1865.8 ppm, respectively. With 10 mg JNM-100-AO, 99.83% of gold can be removed within 5 min, suggesting the ultra-fast adsorption of gold from the e-waste leaching solution (Fig. 6b). Importantly, although Ni^2+^ and Cu^2+^ had much higher concentrations than that of Au^3+^, only 1.26 and 1.08% of Ni^2+^ and Cu^2+^ were adsorbed. The selectivity can be evaluated by considering the relevant *k*_d_ of Au^3+^ (2.94 × 10^6^ mL g^−1^), Ni^2+^ (63.81 mL g^−1^), and Cu^2+^ (54.59 mL g^−1^), suggesting JNM-100-AO is over 4.6 × 10^4^ and 5.4 × 10^4^ folds more selective for Au^3+^ than that for Ni^2+^ and Cu^2+^. Further, the practical application for recovering gold from e-waste was conducted. The leaching solution was obtained from 150 CPUs and the gold concentration was 755.9 ppm, and then JNM-100-AO (250 mg) was added to extract gold, followed by the desorption, reduction and calcination process to produce 0.61 g of gold with a purity of 23.8 Karat (Fig. 6c) (average values of three independent experiments). In contrast, only 0.50 g gold can be obtained under similar procedures using the leaching solutions prepared from aqua regia (gold concentration is 764.9 ppm) (Supplementary Table 10). This might be due to the high concentration of counterions such as Cl^−^ and NO_3_^−^ that compete with the adsorption of [AuCl_4_]^−^.

In summary, we have demonstrated the synthesis of a 2D MOF with vinylene-linkage and Ag(I)-CTUs, namely JNM-100, through Knoevenagel condensation. It can be post-modified with hydroxyl-amine to quantitatively give JNM-100-AO bearing amidoxime functional groups. Owing to the C=C linkage and fully conjugated configuration, JNMs exhibited high stability in a pH range from 0 to 15 and good photoluminescent properties. With high stability and PL properties, JNM-100-AO is able to selectively detect Au^3+^ ions over 20 cations with a low limit of detection of 66 ppb. Interestingly, we have found that JNM-100-AO is an adsorbent for the efficient recovery of gold with high absorption capacity, fast adsorption kinetics, high selectivity, and reusability. More importantly, it can be readily used to rapidly and selectively extract the trace and ultra-trace amounts of gold from complex water mixtures like distilled water with 15 completive metals, leaching solution from e-waste, wastewater, and seawater. Further, the practical application of gold recovery from e-waste was demonstrated with high-purity gold obtained. This work paves the way towards the design novel adsorbents for concentrating ultra-trace amounts of gold from complex liquids.

## Methods

### Synthesis of JNMs

#### JNM-100

Ag_3_L_3_ is synthesized according to our previous reported literature, and 2, 2′,2″-(benzene-1,3,5-triyl)triacetonitrile (1) were purchased and directly used without any purfication^28^. A 10 mL Schlenk tube was charged with Ag_3_L_3_ (45.9 mg, 0.05 mmol), 1 (9.8 mg, 0.05 mmol), mesitylene (0.5 mL), 1,4-dioxane (0.5 mL) and 0.1 mL of 4 M NaOH aqueous. The tube was flash frozen at 77 K in liquid nitrogen bath and degassed with three freeze-pump-thaw cycles. Upon warming to room temperature and ultrasonic microwave treatment for 30 min, and then the mixture was heated at 100 °C for 72 h. The yellow-green solid was isolated by filtration, washed and solvent exchanged with DMF and CH_3_OH. The resultants were dried under vacuum at 100 °C for 8 h to give JNM-100 as green powders (48.3 mg, 82.1% yield). IR (KBr): *ν* = 2235 (m), 1585 (s), 1666 (w), 1540 (s), 1491 (w), 1427 (s), 1373 (w), 1261 (w), 1194 (m), 1035 (s), 1010 (s), 837 cm^−1^ (m).

#### JNM-100-AO

The JNM-100 (400 mg) was immersed in absolute ethanol (40 mL) for 30 min, followed by the addition of NH_2_OH·HCl (1.0 g) and Et_3_N (1.5 g). After stirring at 85 °C for 24 h, the mixture was filtered, washed with excess water and obtained powder were dried at 60 °C under vacuum to give JNM-100-AO as grayish green solid (434.2 mg, 99.1% yield). IR (KBr): *ν* = 1601 (s), 1515 (s), 1420 (w), 1373 (m), 1182 (w), 1035(m), 1011 (m), 841 cm^−1^ (m).

### Adsorption experiments

#### General procedures

In a typical adsorption experiment, 10 mg of JNMs or reference COF (TFPT-BTAN) were mixed with 10 mL of 50 mg L^−1^ Au(III) solution (pH = 2 adjust with concentrated HCl). After reaching adsorption equilibrium, the solid was isolated *via* filtration with 0.22 μm membrane. The filtrate was analyzed using inductively coupled plasma mass spectrometry (ICP-MS) for determination of gold.

#### Adsorption isotherms

The Langmuir model equation is given as Eq. (1):

1 $${q}_{{{{{{\rm{e}}}}}}}=\frac{{C}_{{{{{{\rm{e}}}}}}}{q}_{{{{{{\rm{m}}}}}}}{K}_{{{{{{\rm{L}}}}}}}}{{C}_{{{{{{\rm{e}}}}}}}{K}_{{{{{{\rm{L}}}}}}}+1}$$

where *q*_m_ (mg g^−1^) is the maximum adsorption capacity, *q*_e_ (mg g^−1^) is the equilibrium adsorption capacity, *C*_e_ (mg L^−1^) is the equilibrium concentration of Au(III), *K*_L_ (L mg^−1^) is the Langmuir constant.

#### Adsorption kinetic studies

Ten milligrams of JNMs were mixed with 10 mL of 100 ppm Au(III) solution (pH = 2 adjust with concentrated HCl) for a different time (60−3600 s). The mixture was filtered, and the filtrate was collected and analyzed with ICP-MS to determine the remaining Au(III).

The pseudo-first-order kinetics equation is expressed as Eq. (2):

2 $${{{{{{\rm{ln}}}}}}}(q_{{{{{{\rm{e}}}}}}}-{q}_{{{{{{\rm{t}}}}}}})={{{{{\rm{ln}}}}}}{q}_{{{{{{\rm{e}}}}}}}-{k}_{1}t$$

The pseudo-second-order kinetics equation is shown as Eq. (3):

$$\frac{{{{{{\rm{d}}}}}}{q}_{t}}{{{{{{\rm{d}}}}}}t}={k}_{2}{({q}_{{{{{{\rm{e}}}}}}}-{q}_{{{{{{\rm{t}}}}}}})}^{2}$$

3 $$\frac{{{{{{\rm{t}}}}}}}{{q}_{{{{{{\rm{t}}}}}}}}=\frac{1}{{k}_{2}{q}_{{{{{{\rm{e}}}}}}}^{2}}+\frac{{{{{{\rm{t}}}}}}}{{q}_{{{{{{\rm{e}}}}}}}}$$

where *q*_t_ is the adsorption capacity (mg g^−1^) at a predetermined time t (min) and *q*_e_ is the equilibrium adsorption capacity (mg g^−1^). *k*_1_ (min^−1^) and *k*_2_ (g mg^−1^ min^−1^) is the rate constant of pseudo-first-order and pseudo-second-order adsorption, respectively.

#### Selective adsorption of Au^3+^

The mixed solution (pH = 2 adjust with concentrated HCl) containing Au^3+^ (1 ppm), and 15 competitive metals (50 ppm) including Pt^4+^, Pt^2+^, Ag^+^, Pd^2+^, Fe^3+^, Cu^2+^, Zn^2+^, Ba^2+^, Ni^2+^, Co^2+^, Zr^4+^, Mn^2+^, Al^3+^, Mg^2+^, and Ca^2+^, was prepared. Adding 10 mg of JNMs into 10 mL above as-prepared solution, and soaked for 1 h. After then the mixture was filtrated with 0.22 μm membrane, and the filtrate was analyzed by ICP-MS.

#### Recyclability test

After one run of adsorption, the used JNMs (10 mg) were regenerated by treatment with 30 mL thiourea (1 M, pH = 2 adjust with concentrated HCl) solution and shaken for 5 h, the resulting suspension was filtered and washed with ultra-pure water. The residue was washed with ethanol and dried under vacuum at 80 °C for 6 h to give regenerated JNMs, which were directly used for another adsorption experiment.

#### Gold adsorption from e-waste

Although the e-wastes were commonly pretreated using aqua regia as leaching solution in industry, considering the stability of JNMs in aqua regia and environmental issues, the N-bromosuccinimide (NBS) and pyridine (Py) were used for preparing the leaching solution. It is worthy to mention that the JNMs exhibited lower affinity toward gold in aqua regia system than that in NBS/Py (See SI for details) due to high concentration of counterions such as Cl^−^ and NO_3_^−^ that compete with the adsorption of [AuCl_4_]^−^. The leaching solution was prepared by mixing 100 mL of distilled water with 20 μL of pyridine (5 M) and NBS (360 mg, 18 mmol) (See SI for more details). The 3 CPU processors were treated with leaching solution at rt for 24 h, and the mixture was acidified to pH = 2 with concentrated HCl (Au^3+^ concentration ~105 ppm analyzed by ICP-MS). After then above prepared leaching solution (8 mL) was diluted with water (92 mL), the mixture was subsequently analyzed by ICP-MS to determine the metal concentrations (Au^3+^ 8.5 ppm, Ni^2+^ 1468.2 ppm and Cu^2+^ 1865.8 ppm). After that, 10 mg of JNMs were added to 50 mL above prepared leaching solution at rt for 1 h, then the mixture was filtrated with 0.22 μm membrane. The filtrate was analyzed with ICP-MS for determination of elemental concentration.

The distribution coefficient (*k*_d_) value as used for the determination of the affinity and selectivity of sorbents for Au(III) (mL g^−1^), is given by the equation as Eq. (4):

4 $${k}_{{{{{\rm{d}}}}}}=\frac{({C}_{0}-{C}_{{{{{\rm{e}}}}}})}{{C}_{{{{{\rm{e}}}}}}}\times \frac{V}{m}$$

Where V is the volume of the treated solution (mL), m is the amount of used adsorbent (g), and *C*_0_ and *C*_e_ are the initial concentration and the final equilibrium concentration of Au (III) (mg L^−1^), respectively. Herein, 10 mg of JNMs was added to 50 mL leaching solution, thus *V*/*m* = 5000 mL g^−1^.

#### Practical application of gold recovery from e-waste treated with NBS/Py

The leaching solution was prepared by mixing 500 mL of distilled water with 50 mL of pyridine (5 M) and NBS (25 g). The 150 CPUs was added to above prepared mixture to give the metal leaching solution. After then, 250 mg of JNM-100-AO added to the leaching solution, stirred for 24 hours, and finally filter to obtain a gray brown powder JNM-100-AO-Au. The resulted powder was soaking in 100 mL 1 M thiourea solution (pH = 2 adjust with concentrated HCl) and stirred 5 hours to give yellow solution and JNM-100-AO. After filtration, the JNM-100-AO was added again into leaching solution to collect residual gold, then desorbed with thiourea solution. Such processes were repeated three times. The resulted yellow solution was reduced with Na_2_S_2_O_5_ to give a black powder of gold. The powder was washed three times with 50 mL water and then air-dried. Then, 10 mg of borax was mixed into the powder as a stabilizer and the mixture was sintered at high temperature, and a molten golden yellow solid was obtained. The obtained gold was weighed, and 1 mg of gold was scraped and dissolved in 10 mL of aqua regia, and the purity of gold was determined by ICP-MS. It is necessary to mention that the thiourea in acid solution exhibited higher affinity to gold than that copper and nickel^42^.

### DFT calculations

All calculations were performed by using the density functional method conducted out by the Cambridge Sequential Total Energy Package (CASTEP)^43^ module embedded in Materials Studio 2018 (MS 2018). The Perdew-Burke-Ernzerhof functional^44^ of GGA was employed in cell relaxation and geometry optimization. Protons were randomly added to the vacuum layer of the computational model to simulate the effect of counterions under acidic conditions. The OTFG ultra-soft pseudopotentials^45^ were used to describe all atoms with an energy cutoff of 400 eV. The total energy difference and maximum residual force were converged within 10^−5^ eV and 0.05 eV/Å during optimization. The k-point mesh of 1 × 1 × 1 was generated by Monkhorst-Pack scheme^46^. The JNM-100 monolayer for the adsorption simulation was modeled using a 1 × 1 slab (~25 Å × 25 Å) repeated periodically along the x- and y- directions and a vacuum space up to 50 Å was applied along the z-direction to avoid the interaction between two neighboring images. The reference COF, TFPT-BTAN, is also modeling with the same method.

The binding energy (see Eq. (5)) is defined as the difference between the total energy of the adsorption system and the energy sum of isolated adsorbents including JNM-100 or TFPT-BTAN monolayer and [AuCl_4_]^−^ ions:

5 $${E}_{{{{{\rm{b}}}}}}={E}_{{{{{{\rm{total}}}}}}}-{E}_{{{{{{\rm{absorbents}}}}}}}-{E}_{{[{{{{{\rm{AuCl}}}}}}_{4}]}^{-}}$$

The absolute value determines the strength of the action, and the sign of the value determines whether the action is attraction or repulsion.

## Supplementary information

Supplementary Information

## Data Availability

The data that support the findings of this study are available within the paper and its supplementary information files or are available from the corresponding authors upon request.
