# Supplementary material for: Selective and rapid extraction of trace amount of gold from complex liquids with silver(I)-organic frameworks
Source: Nat Commun. 2022 Dec 15;13:7771. doi: 10.1038/s41467-022-35467-z (PMC9755257; doi:10.1038/s41467-022-35467-z)
Supplement: Supplementary file 1 — Supplementary Information [file 41467_2022_35467_MOESM1_ESM.pdf]

Supplementary Information

Selective and Rapid Extraction of Trace Amount of Gold from Complex Liquids with Silver(I)-Organic Frameworks

Jie Luo,[1] Xiao Luo,[1] Mo Xie,[1] Hao-Zhen Li,[1] Hai-Yan Duan,[1] Hou-Gan Zhou,[1] Rong-Jia Wei,[1] Guo-Hong Ning,[1,*] and Dan Li[1,*]

[1] College of Chemistry and Materials Science, and Guangdong Provincial Key Laboratory of Functional Supramolecular Coordination Materials and Applications, Jinan University, Guangzhou 510632

E-mail addresses:
guohongning@jnu.edu.cn (G.-H. Ning)
danli@jnu.edu.cn (D. Li)

Contents

1. General procedure

2. Powder X-ray diffraction (PXRD)

3. Fourier-transform infrared (FT-IR) spectra

4. Solid-state ^{13}C CP/MAS NMR spectra

5. Scanning electron microscopy (SEM)

6. Transmission electron microscopy (TEM)

7. Energy Dispersive X-ray Spectroscopy (EDS)

8. Thermogravimetric analysis (TGA)

9. Various-Temperature PXRD

10. Stability in various solvents

11. Crystal structure modeling

12. Photoluminescent properties

13. Detection of gold

14. Adsorption of gold

15. Extraction of gold from e-waste

16. XPS spectra

17. DFT calculations

1. General procedure

1.1. Instruments and methods.

All the chemicals were commercially available and used without further purification. Powder X-ray diffraction (PXRD) data was collected at 40kV, 30 mA using microcrystalline samples on a Rigaku Ultima IV diffractometer using Cu-Kα radiation (λ = 1.5418 Å). The measurement parameters include a scan speed of 0.5°/min, a step size of 0.02°, and a scan range of 2θ from 2.5° to 30°. For Temperature-dependent PXRD, the measurement parameters include a scan speed of 2 °C/min, a step size of 0.02°, and a scan range of 2θ from 1.5° to 30°.Thermogravimetric analysis was performed on a Mettler-Toledo (TGA/DSC1) thermal analyzer. Measurement was made on approximately 5 mg of dried samples under a N_2 flow with a heating rate of 10 °C/min. The scanning electron microscopy (SEM) images and Energy Dispersive X-ray Spectroscopy (EDS) were obtained on a JEOL JSM7600F microscope, operating at an acceleration voltage of 5 kV and magnification is 2000.Transmission electron microscopy (TEM) analysis was performed on a FEI Titan 80-300 S/TEM (Scanning /Transmission Electron Microscope) operated at 200 kV. Fourier transform infrared (FT-IR) spectrum was measured using a Nicolet Avatar 360 FT-IR spectrophotometer. X-ray photoelectron (XPS) spectroscopy spectra were performed by a Thermo ESCALAB 250XI system. Liquid ^{1}H and ^{13}CNMR spectra were recorded on a Bruker Biospin Avance (400 MHz) equipment using tetramethylsilane (TMS) as an internal standard. Solid-state NMR experiments were performed on a Bruker WB Avance II 600 MHz NMR spectrometer. The ^{13}C CP/MAS NMR spectra were recorded with a 4-mm double-resonance MAS probe and with a sample spinning rate of 10.0 kHz; a contact time of 2 ms (ramp 100) and a pulse delay of 3 s was applied. Gas sorption analyses were conducted using an ASAP 2020 PLUS Analyzer (Micromeritics) with extra-high pure gases. The samples were outgassed at 120 ºC for 12 h before the measurements. Surface areas were calculated from the adsorption data using Brunauer-Emmett-Teller (BET) methods. The pore size distribution curves were obtained from the adsorption branches using density functional theory (DFT) method. Inductively coupled plasma mass spectrometry (ICP-MS) tests were performed on Agilent 7900 ICP-MS instrument.

1.2 Synthesis of JNM-100 and JNM-100-AO

Ag_3L_3 is synthesized according to our previous reported literature (See manuscript ref. 27). In a 25 mL flask, 20 mg (0.1 mmol) 4-(3,5-dimethyl-1H-pyrazol-4-yl)benzaldehyde (HL), 3 mL tetrahydrofuran and 100 μL triethylamine were added. After stirring for 5 min, 22.9 mg (0.1 mmol) PhCOOAg was added. After stirring in the dark for 12 hours, filtered and washed with ethanol to obtain 12.3 mg of white solid, with a yield of 40.05%. ^{1}H NMR (400 MHz, DMSO-d_6, δ) 9.99 (s, 1H), 7.91 (d, J = 8.3 Hz, 2H), 7.52 (d, J = 8.1 Hz, 2H), 2.33 (s, 6H). IR (KBr): v = 2950 (w), 2913 (w), 2820 (w), 2718 (w), 1698 (s), 1601 (s), 1562 (s), 1538 (s), 1489 (s), 1424 (s), 1387 (w), 1337 (w), 1305 (m), 1215 (s), 1169 (s), 1114 (w), 1027 (s), 838 (s), 729 (w), 698 (w), 659 (w), 603 (w), 562 (m), 519 cm^{-1} (m).

JNM-100. 2,2',2''-(benzene-1,3,5-triyl) triacetonitrile (**1**) were purchased from Jilin Chinese Academy of Science-Yan shen Technology Co., Ltd and directly used without any purification. A 10 mL Schlenk tube was charged with Ag_3L_3 (45.9 mg, 0.05 mmol), **1** (9.8 mg, 0.05 mmol), mesitylene (0.5 mL), dioxane (0.5 mL) and 0.1 mL of 4 M NaOH aqueous. The tube was flash frozen at 77 K in liquid nitrogen bath and degassed with three freeze-pump-thaw cycles. Upon warming to room temperature and ultrasonic microwave treatment for 30 min, and then the mixture was heated at 100 °C for 72 h. The yellow-green solid was isolated by filtration, washed and solvent exchanged with DMF and CH_3OH. The resultants were dried under vacuum at 100 °C for 8 h to give JNM-100 as green powders (48.3 mg, 82.1% yield). IR (KBr): v = 2235 (m), 1585 (s), 1666 (w), 1540 (s), 1491 (w), 1427 (s), 1373 (w), 1261 (w), 1194 (m), 1035 (s), 1010 (s), 837 cm^{-1} (m).

JNM-100-AO. The JNM-100 (400 mg) was immersed in absolute ethanol (40 mL) for 30 min, followed by the addition of $NH_2OH{\cdot}HCl$ (1.0 g) and $N(CH_2CH_3)_3$ (1.5 g). After stirring at 85 °C for 24 h, the mixture was filtered, washed with excess water and obtained powder were dried at 60 °C under vacuum to give JNM-100-AO as grayish green solid (434.2 mg, 99.1% yield). IR (KBr): v = 1601 (s), 1515 (s), 1420 (w), 1373 (m), 1182 (w), 1035(m), 1011 (m), 841 cm^{-1} (m).

1.3 Synthesis of reference COF material, TFPT-BTAN.

Synthesis of TFPT-BTAN. TFPT-BTAN is synthesized according to reported literature.[1] To a 25 mL Pyrex tube, 2,4,6-tris(4-formylphenyl)-1,3,5-triazine (78.68 mg, 0.20 mmol), 2,2′,2″-(benzene-1,3,5-triyl)tria-cetonitrile (39.04 mg, 0.20 mmol), *o*-DCB (5 mL) and DBU aqueous solution (0.5 mL, 4 M) were added. The mixture was sonicated for 10 minutes, degassed by three freeze–pump–thaw cycles, sealed under vacuum and heated at 90 °C for 5 days. The reaction mixture was cooled to room temperature, and a pale-yellow precipitate was collected by centrifugation, washed several times with methanol, CH_2Cl_2, and THF, respectively. It was then Soxhlet extracted in CH_2Cl_2 and THF for 24 h and dried under vacuum at 80 °C for 12 h to afford pale-yellow powder, 67% yield. IR(KBr): v = 2250 (m), 1680 (s), 1574 (m), 1508 (s), 1420 (w), 1360 (s), 1250 (m), 1110 (m), 820 (s), 780 cm^{-1} (s).

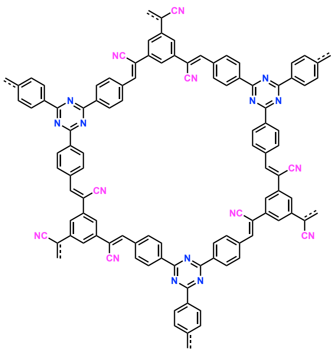

TFPT-BTAN

2. Powder X-ray diffraction (PXRD)

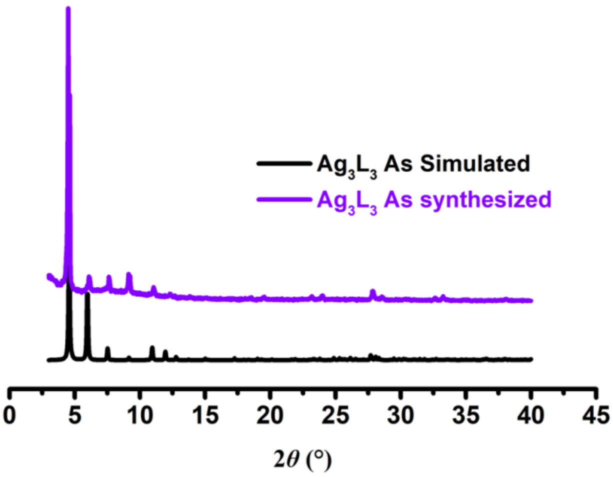

Supplementary Figure 1. The PXRD of Ag_3L_3, confirming the purity of Ag_3L_3 powder.

3. Fourier-transform infrared (FT-IR) spectra

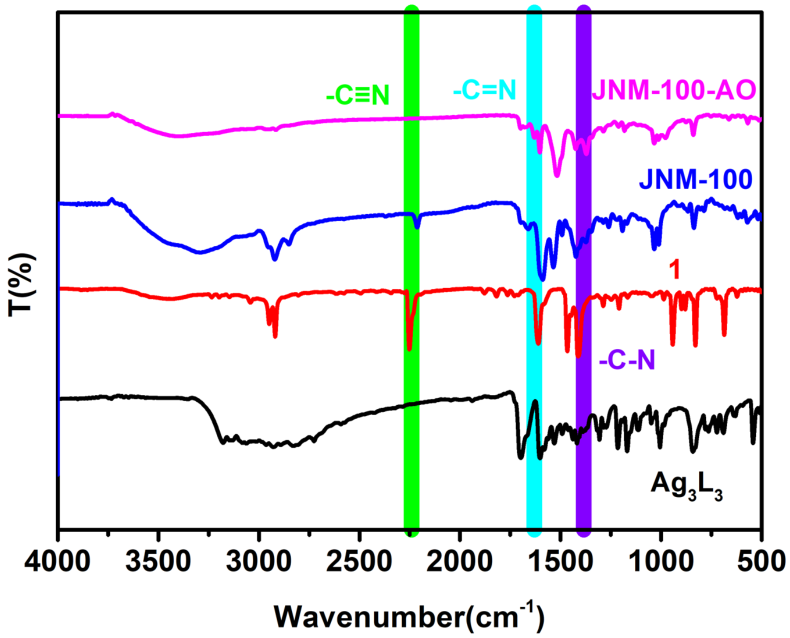

Supplementary Figure 2. FT-IR of JNM-100 (Blue), JNM-100-AO (Pink) and all the ligands (Red and Black).

4. Solid-state ^{13}C CP/MAS NMR spectra

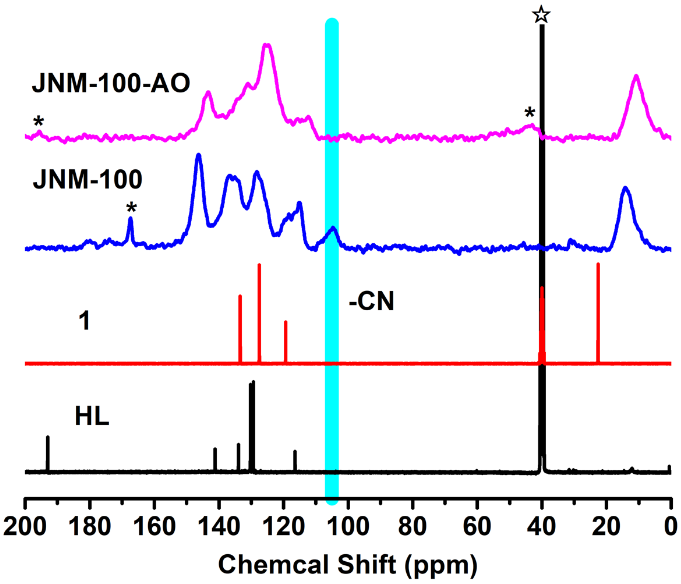

Supplementary Figure 3. Solid-state ^{13}C CP/MAS NMR (100 MHz, 300 K) spectra of JNM-100 (Blue) and JNM-100-AO (Pink), indigo ribbon at 105 ppm is the peak of -CN, and ^{13}C NMR (100 MHz, 300 K, DMSO) spectra of linker **1** (red) and ligand HL (black) (☆mark represents *d*6-DMSO, * mark represents spin sideband).

5. Scanning electron microscopy (SEM)

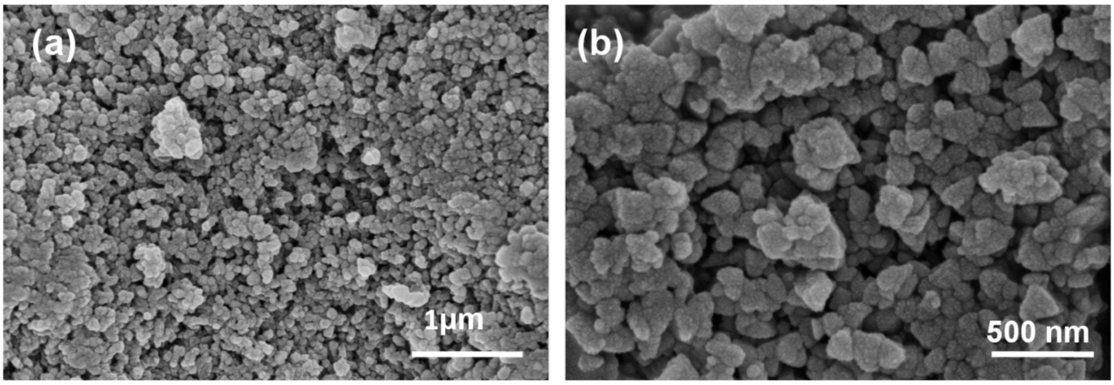

Supplementary Figure 4. SEM images of JNM-100. (a) JNM-100 in 1 μm scale, and (b) in 500 nm scale.

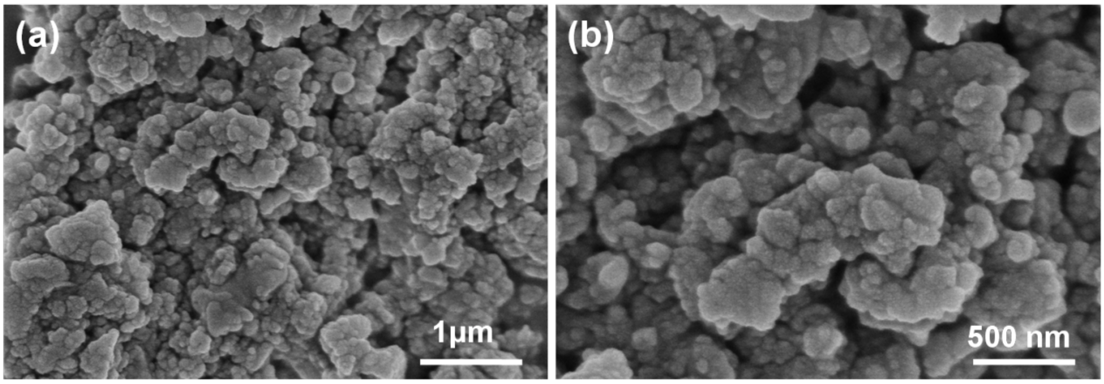

Supplementary Figure 5. SEM images of JNM-100-AO. (a) JNM-100-AO in 1 μm scale, and (b) in 500 nm scale.

6. Transmission electron microscopy (TEM)

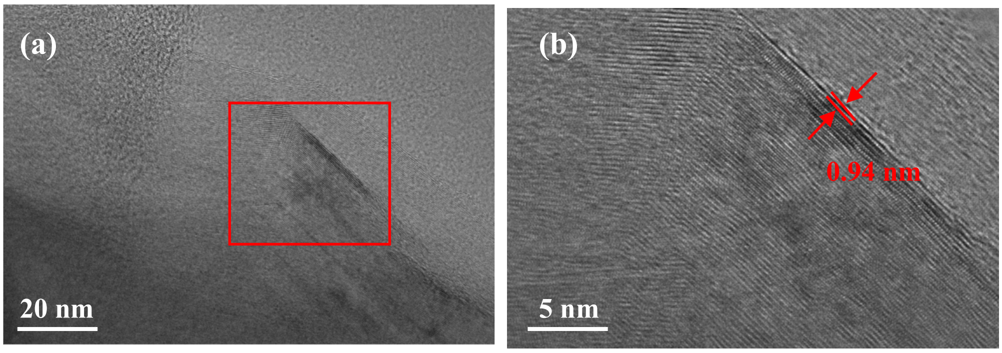

Supplementary Figure 6. TEM images of JNM-100. (a) JNM-100 in 20 nm scale, and (b) zoom-in TEM view in 5 nm scale.

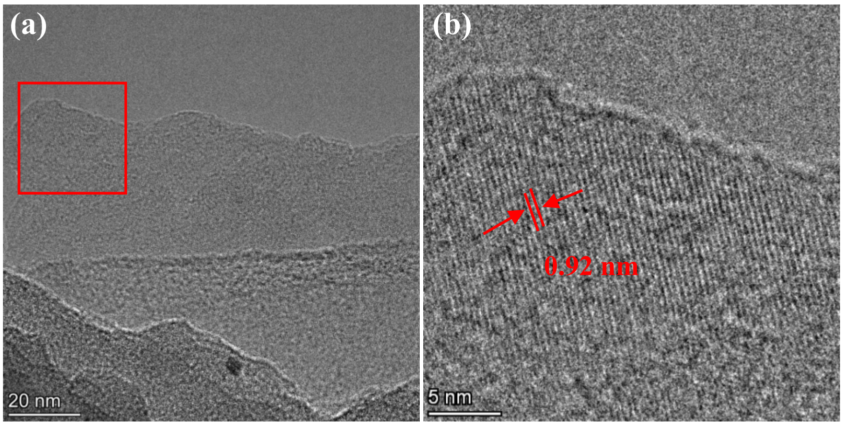

Supplementary Figure 7. TEM images of JNM-100-AO. (a) JNM-100-AO in 20 nm scale, and (b) zoom-in TEM view in 5 nm scale.

7. Energy Dispersive X-ray Spectroscopy (EDS)

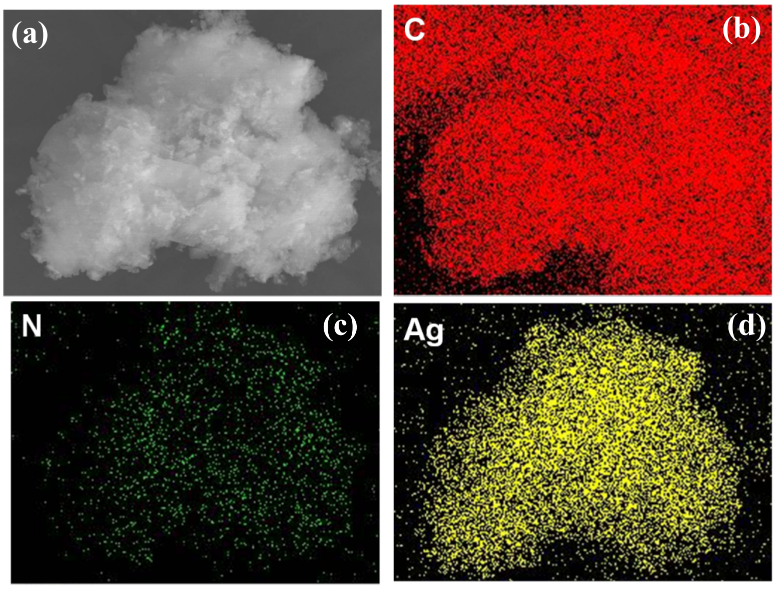

Supplementary Figure 8. EDS of JNM-100. (a) SEM photo of JNM-100; Element mapping of (b) C: Red; (c) N: Green; and (d) Ag: Yellow.

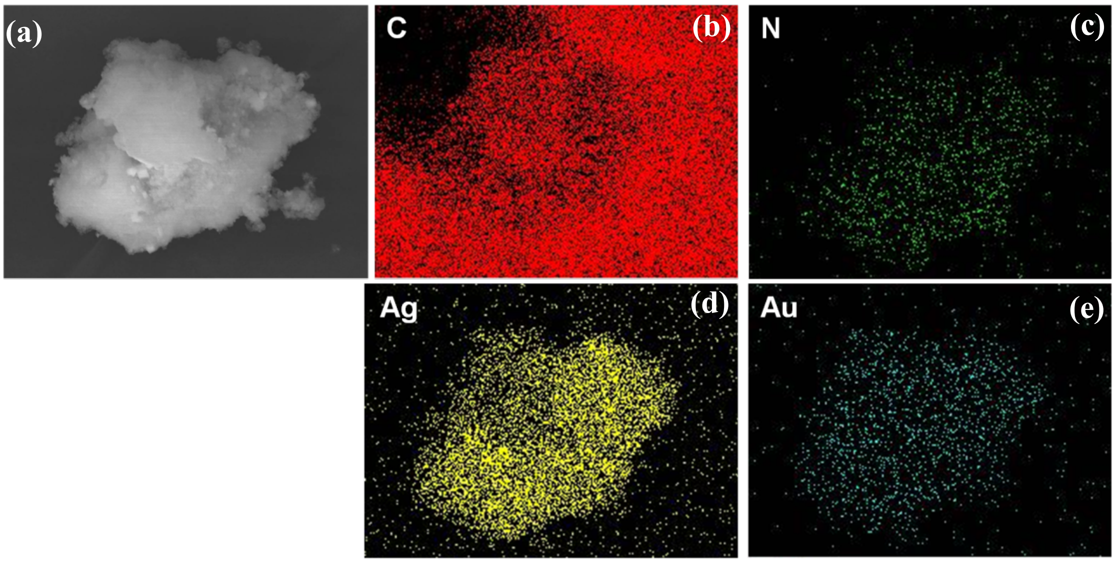

Supplementary Figure 9. EDS of **JNM-100-Au(III)**. (a) SEM photo of **JNM-100-Au(III)**; Element mapping of (b) C: Red; (c) N: Green; (d) Ag: Yellow; and (e) Au: Indigo .

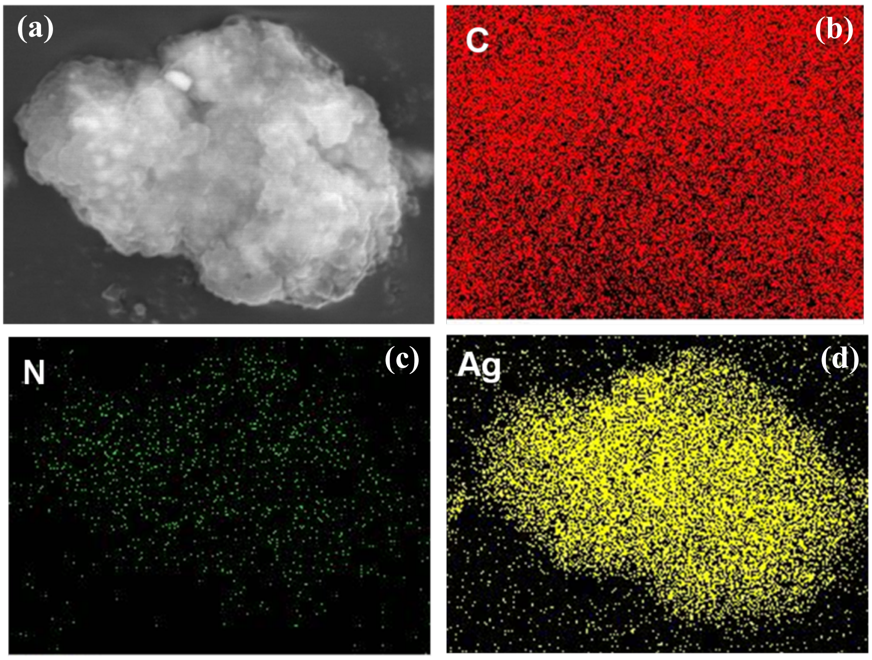

Supplementary Figure 10. EDS of JNM-100-AO. (a) SEM photo of JNM-100-AO; Element mapping of (b) C: Red; (c) N: Green; and (d) Ag: Yellow.

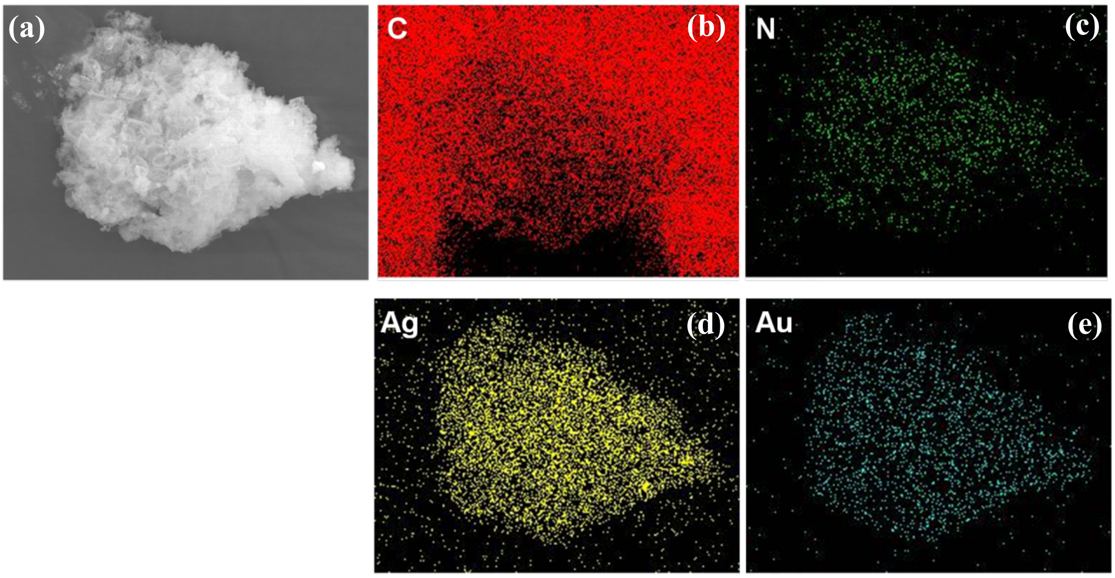

Supplementary Figure 11. EDS of JNM-100-AO-Au(III). (a) SEM photo of JNM-100-AO-Au(III); Element mapping of (b) C: Red; (c) N: Green; (d) Ag: Yellow; and (e) Au: Indigo .

8. Thermogravimetric analysis (TGA)

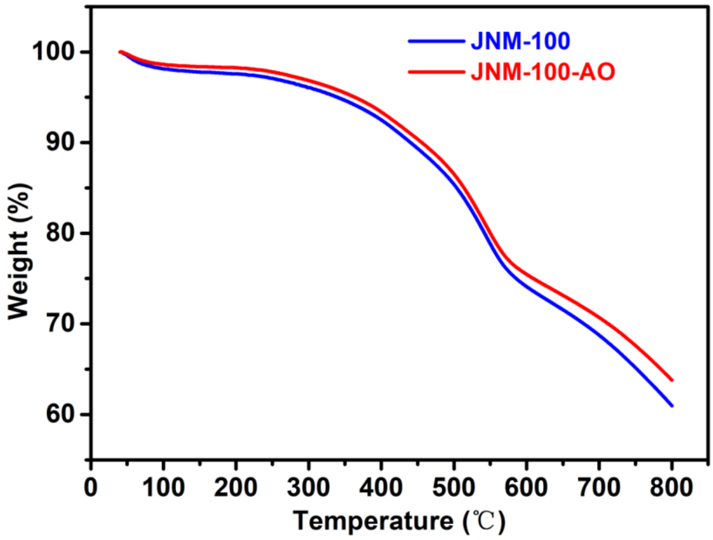

Supplementary Figure 12. TGA curves of JNM-100 (Blue) and JNM-100-AO (Red) under the N_2 atmosphere.

9. Various-Temperature PXRD

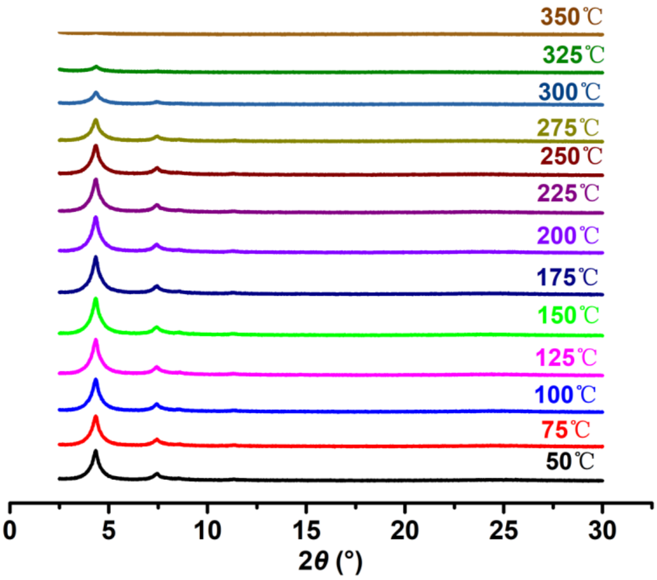

Supplementary Figure 13. *In-situ* variable-temperature PXRD patterns of JNM-100 under the N_2 atmosphere.

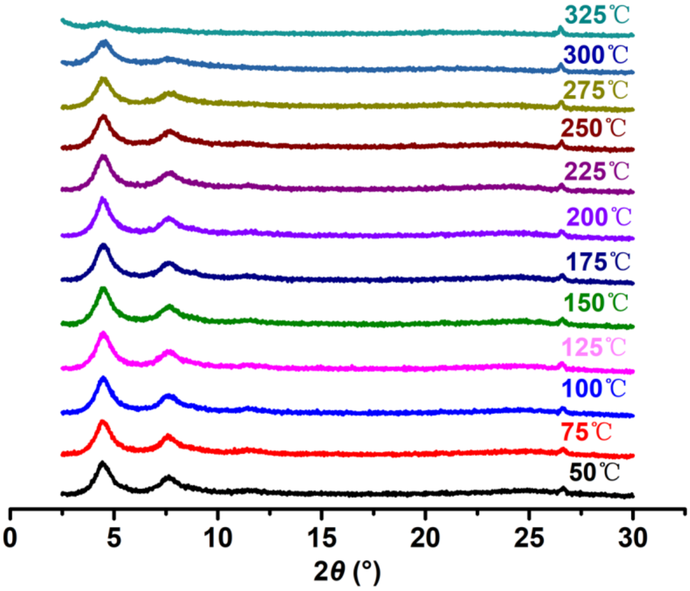

Supplementary Figure 14. *In-situ* variable-temperature PXRD patterns of JNM-100-AO under the N_2 atmosphere.

10. Stability in various solvents

JNM-100 or JNM-100-AO (10 mg) was added to a 20 mL glass vial containing 10 mL solution including water, dioxane, methanol, chloroform, DMF, DMSO, 10 M NaOH solution and 1 M HCl solution. The mixture was stand at room temperature for 3 days, after then the JNMs powders were filtrated and vacuum dried at 100 °C for 12 h. The obtained powder was weighed and tested by PXRD and the concentration of silver ions in the filtrate was determined by ICP-MS. Considering the application of gold adsorption, the N_2 absorption profiles of samples that after immersing in 1 M HCl, and after 5 cycles of absorption of gold, were recorded.

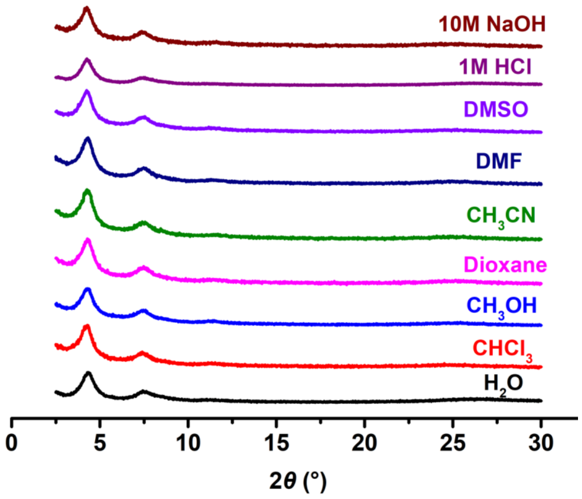

Supplementary Figure 15. PXRD patterns for 10 mg of JNM-100 after treatment with different solvents for 3 days.

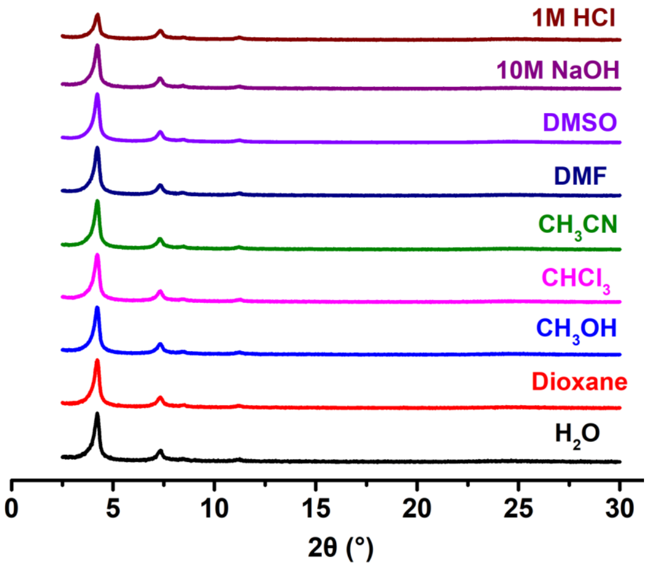

Supplementary Figure 16. PXRD patterns for 10 mg of JNM-100-AO after treatment with different solvents for 3 days.

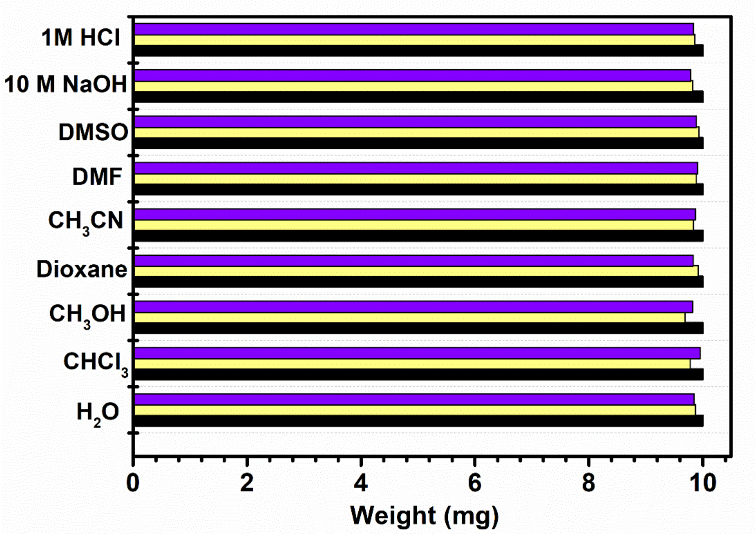

Supplementary Figure 17. Residual weight of JNMs after treatment in various solvent. (Black: initial weight, JNM-100 yellow, and JNM-100-AO purple)

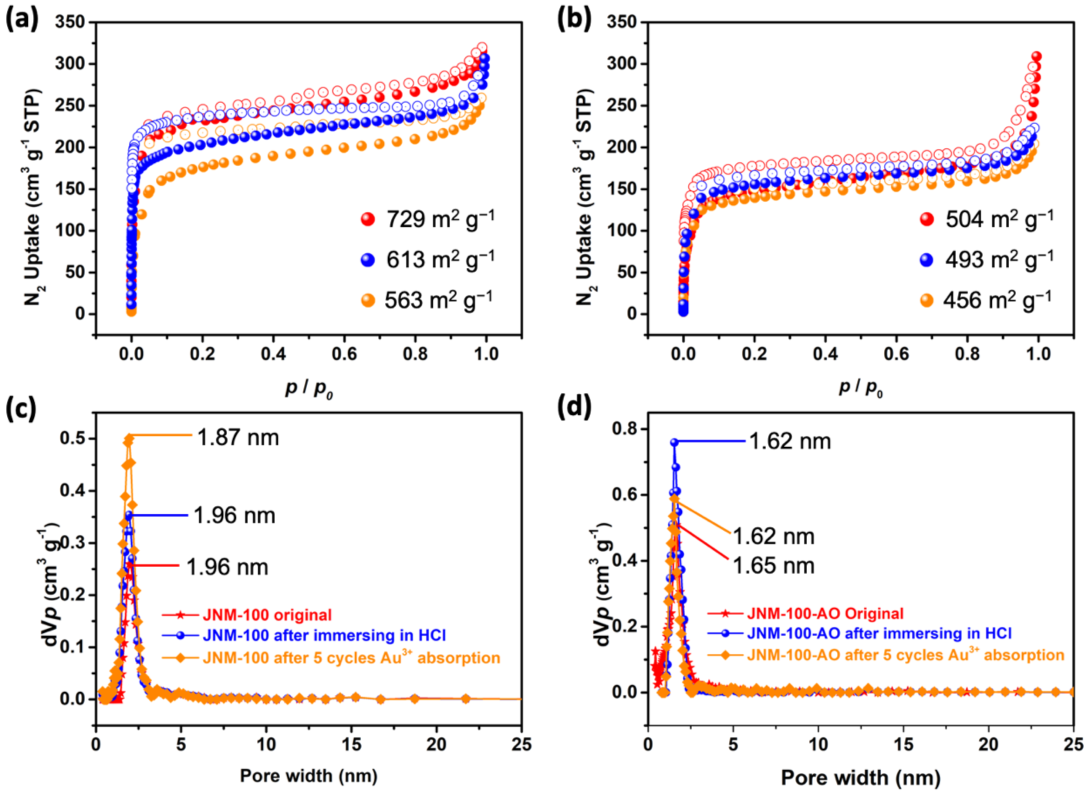

Supplementary Figure 18. The surface area analysis of JNMs. (a) the N_2 absorption profiles of JNM-100 (red), after immersing in 1M HCl (blue), and after 5 cycles of absorption of gold (orange); (b) the N_2 absorption profiles of JNM-100-AO (red), after immersing in HCl (blue), and after 5 cycles of absorption of gold (orange); (c) the pores size distribution of JNM-100 (red), after immersing in 1M HCl (blue), and after 5 cycles of absorption of gold (orange); (d) the pores size distribution of JNM-100-AO (red), after immersing in 1M HCl (blue), and after 5 cycles of absorption of gold (orange);

10. Crystal structure modeling

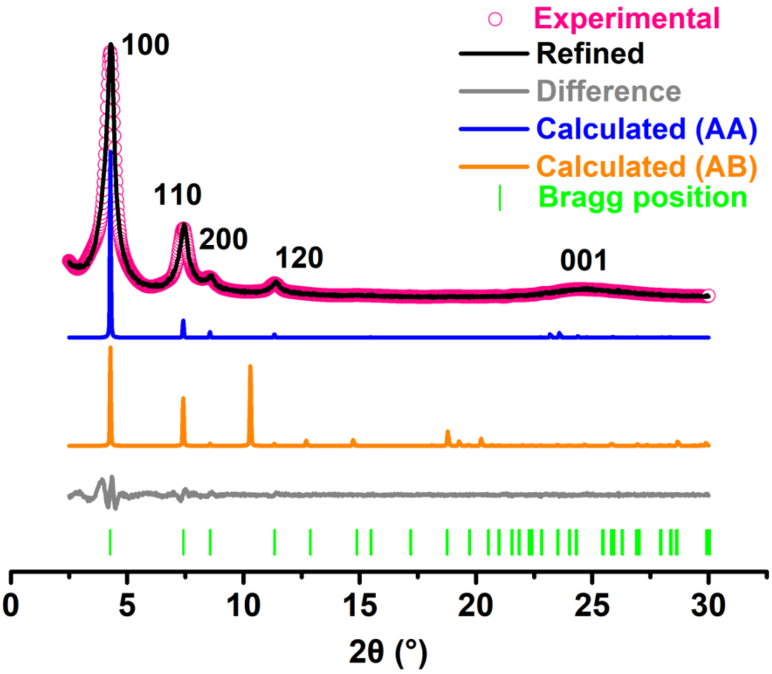

Supplementary Figure 19. JNM-100 refinement of the eclipsed structural model by the measured PXRD pattern using the Pawley method.

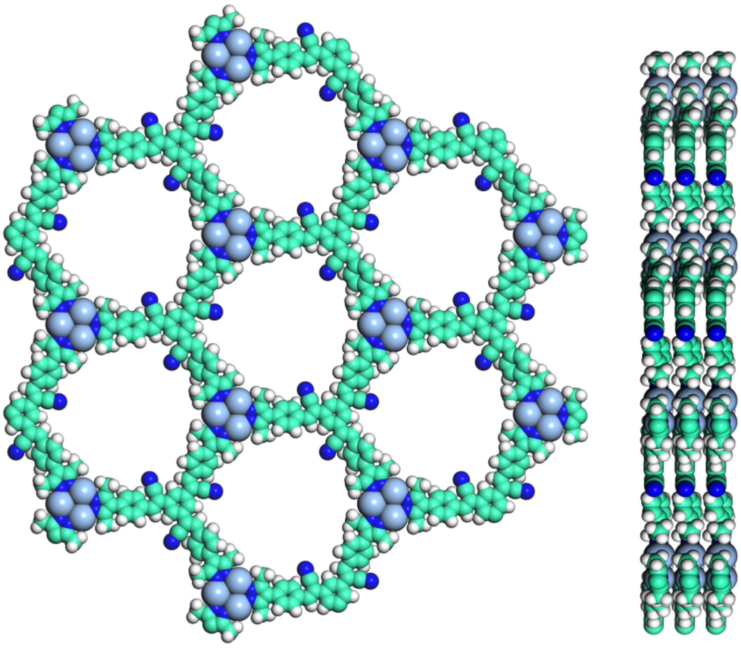

Supplementary Figure 20. Space-filling mode of JNM-100 in AA stacking model viewed from *c* axis(left) and *a* axis(right).

Supplementary Table 1. AA stacking model for JNM-100

Hexagonal*P*-3<br>*a* = *b* = 23.9030 Å, *c* = 3.8141 Å			
Ag	0.57149	0.2887	0.47128
N	0.58009	0.37506	0.47874
N	0.63392	0.42671	0.4655
N	0.29774	0.46179	0.68995
C	0.3542	0.62323	0.54198
C	0.29058	0.60332	0.54089
C	0.37597	0.57722	0.54756
C	0.53463	0.38899	0.48901
C	0.62438	0.47512	0.46108
C	0.5608	0.45324	0.47705
C	0.52844	0.48969	0.48119
C	0.47021	0.46658	0.32205
C	0.43933	0.50028	0.33037
C	0.46625	0.55813	0.49682
C	0.52513	0.58233	0.64482
C	0.55549	0.5481	0.64248
C	0.4036	0.83994	0.50448
C	0.33256	0.51305	0.62641
C	0.67589	0.54033	0.41907
C	0.46813	0.3409	0.53605
H	0.44904	0.42327	0.18156
H	0.39556	0.48192	0.19548
H	0.54699	0.62718	0.77147
H	0.59942	0.56703	0.77479
H	0.25748	0.5543	0.54058
H	2.90688	3.33939	3.26114
H	2.82525	3.28446	3.28438
H	2.87051	3.3082	3.67866
H	2.88055	3.55476	3.27807
H	2.91548	3.55627	3.69513
H	2.83524	3.53524	3.67197

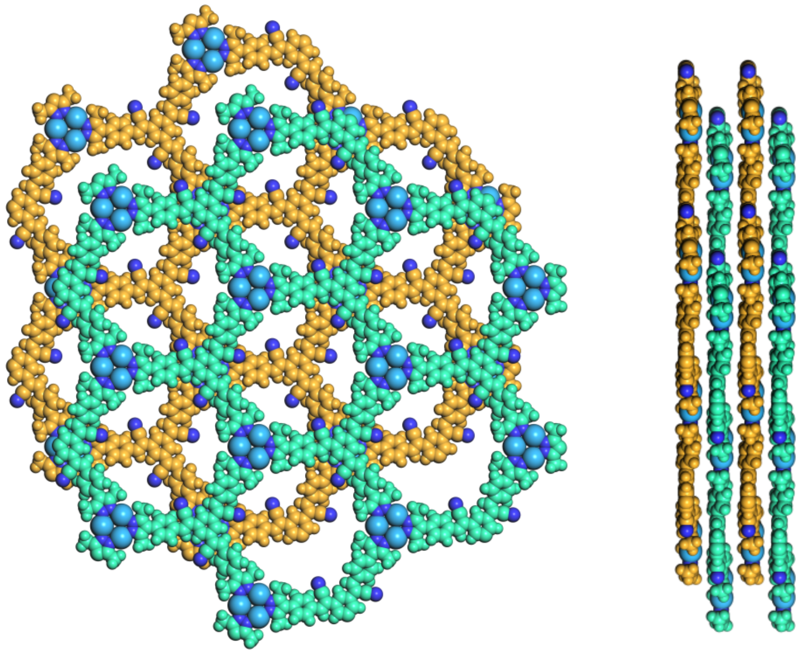

Supplementary Figure 21. Space-filling mode of JNM-100 in AB stacking model viewed from *c* axis(left) and *a* axis(right).

Supplementary Table 2. AB stacking model for JNM-100.

Hexagonal*P*-3											
a = *b* = 23.9030 Å, *c* = 9.3781 Å											
Ag	0.90479	0.9554	0.24579	C	0.7852	0.67408	0.31541	H	0.26612	0.90033	0.86923
Ag	0.23819	0.622	0.74579	C	0.49336	0.23036	0.25929	H	0.92418	0.8876	0.77397
Ag	0.0505	0.09521	0.24579	C	0.82025	0.48621	0.30888	H	0.57358	0.67269	0.66032
Ag	0.378	0.6161	0.74579	C	0.79297	0.80226	0.22455	H	0.49195	0.61776	0.66977
Ag	0.3839	0.76181	0.74579	C	0.9924	0.79393	0.27213	H	0.53721	0.6415	0.83013
Ag	0.0446	0.9495	0.24579	H	0.91003	0.69248	0.12796	H	0.54725	0.88806	0.66721
N	0.91339	0.04176	0.24882	H	0.85138	0.58034	0.13362	H	0.58218	0.88957	0.83683
N	0.63104	0.12849	0.33472	H	0.70612	0.58651	0.36788	H	0.50194	0.86854	0.82741
N	0.95824	0.87173	0.24882	H	0.76627	0.69909	0.36923	C	0.04347	0.06426	0.77454
N	0.90659	0.87391	0.24344	H	0.779	0.36988	0.27397	C	0.06338	0.02056	0.7741
N	0.83075	0.03556	0.83472	H	0.99391	0.23418	0.16032	C	0.08948	0.13205	0.77681
N	0.46167	0.75321	0.74882	H	0.04884	0.20749	0.16977	C	0.27771	0.47895	0.753
N	0.45949	0.69938	0.74344	H	0.0251	0.229	0.33013	C	0.19158	0.48256	0.74164
N	0.20491	0.16925	0.83472	H	0.77854	0.99249	0.16721	C	0.21346	0.44087	0.74813
N	0.49735	0.36896	0.33472	H	0.77703	0.02591	0.33683	C	0.17701	0.37205	0.74982
N	0.87151	0.50265	0.33472	H	0.79806	0.9667	0.32741	C	0.20012	0.33693	0.68509
N	0.12827	0.08661	0.24882	C	0.60234	0.3125	0.27454	C	0.16642	0.27235	0.68848
N	0.24679	0.70836	0.74882	C	0.64604	0.37612	0.2741	C	0.10857	0.24142	0.75617
N	0.30062	0.76001	0.74344	C	0.53455	0.29073	0.27681	C	0.08437	0.27611	0.81637
N	0.12609	0.03278	0.24344	C	0.18765	0.13207	0.253	C	0.1186	0.34068	0.81541
N	0.29164	0.53833	0.74882	C	0.18404	0.04232	0.24164	C	0.82676	0.89696	0.75929
N	0.23999	0.54051	0.74344	C	0.22573	0.1059	0.24813	C	0.15365	0.15281	0.80888
N	0.96444	0.79509	0.83472	C	0.29455	0.13826	0.24982	C	0.12637	0.46886	0.72455
N	0.96722	0.09341	0.24344	C	0.32967	0.19649	0.18509	C	0.3258	0.46053	0.77213
C	0.6875	0.28993	0.27454	C	0.39425	0.22737	0.18848	H	0.24343	0.35908	0.62796
C	0.62388	0.27002	0.2741	C	0.42518	0.20045	0.25617	H	0.18478	0.24694	0.63362
C	0.70927	0.24392	0.27681	C	0.39049	0.14157	0.31637	H	0.03952	0.25311	0.86788

C	0.86793	0.05569	0.253	C	0.32592	0.11121	0.31541	H	0.09967	0.36569	0.86923
C	0.95768	0.14182	0.24164	C	0.76964	0.2631	0.25929	H	0.1124	0.03648	0.77397
C	0.8941	0.11994	0.24813	C	0.51379	0.33414	0.30888	H	0.32731	0.90078	0.66032
C	0.86174	0.15639	0.24982	C	0.19774	0.99081	0.22455	H	0.38224	0.87409	0.66977
C	0.80351	0.13328	0.18509	C	0.20607	0.19857	0.27213	H	0.3585	0.8956	0.83013
C	0.77263	0.16698	0.18848	H	0.30752	0.21766	0.12796	H	0.11194	0.65909	0.66721
C	0.79955	0.22483	0.25617	H	0.41966	0.27114	0.13362	H	0.11043	0.69251	0.83683
C	0.85843	0.24903	0.31637	H	0.41349	0.11971	0.36788	H	0.13146	0.6333	0.82741
C	0.88879	0.2148	0.31541	H	0.30091	0.06728	0.36923	C	0.93574	0.9791	0.77454
C	0.7369	0.50664	0.25929	H	0.63012	0.40922	0.27397	C	0.97944	0.04272	0.7741
C	0.66586	0.17975	0.30888	H	0.76582	0.75982	0.16032	C	0.86795	0.95733	0.77681
C	0.00919	0.20703	0.22455	H	0.79251	0.84145	0.16977	C	0.52105	0.79867	0.753
C	0.80143	0.0076	0.27213	H	0.771	0.79619	0.33013	C	0.51744	0.70892	0.74164
H	0.78234	0.08997	0.12796	H	0.00751	0.78615	0.16721	C	0.55913	0.7725	0.74813
H	0.72886	0.14862	0.13362	H	0.97409	0.75122	0.33683	C	0.62795	0.80486	0.74982
H	0.88029	0.29388	0.36788	H	0.0333	0.83146	0.32741	C	0.66307	0.86309	0.68509
H	0.93272	0.23373	0.36923	C	0.0209	0.95653	0.77454	C	0.72765	0.89397	0.68848
H	0.59078	0.221	0.27397	C	0.95728	0.93662	0.7741	C	0.75858	0.86705	0.75617
H	0.24018	0.00609	0.16032	C	0.04267	0.91052	0.77681	C	0.72389	0.80817	0.81637
H	0.15855	0.95116	0.16977	C	0.20133	0.72229	0.753	C	0.65932	0.77781	0.81541
H	0.20381	0.9749	0.33013	C	0.29108	0.80842	0.74164	C	0.10304	0.9297	0.75929
H	0.21385	0.22146	0.16721	C	0.2275	0.78654	0.74813	C	0.84719	0.00074	0.80888
H	0.24878	0.22297	0.33683	C	0.19514	0.82299	0.74982	C	0.53114	0.65741	0.72455
H	0.16854	0.20194	0.32741	C	0.13691	0.79988	0.68509	C	0.53947	0.86517	0.77213
C	0.71007	0.39766	0.27454	C	0.10603	0.83358	0.68848	H	0.64092	0.88426	0.62796
C	0.72998	0.35396	0.2741	C	0.13295	0.89143	0.75617	H	0.75306	0.93774	0.63362
C	0.75608	0.46545	0.27681	C	0.19183	0.91563	0.81637	H	0.74689	0.78631	0.86788
C	0.94431	0.81235	0.253	C	0.22219	0.8814	0.81541	H	0.63431	0.73388	0.86923
C	0.85818	0.81596	0.24164	C	0.0703	0.17324	0.75929	H	0.96352	0.07582	0.77397
C	0.88006	0.77427	0.24813	C	0.99926	0.84635	0.80888	H	0.09922	0.42642	0.66032
C	0.84361	0.70545	0.24982	C	0.34259	0.87363	0.72455	H	0.12591	0.50805	0.66977
C	0.86672	0.67033	0.18509	C	0.13483	0.6742	0.77213	H	0.1044	0.46279	0.83013
C	0.83302	0.60575	0.18848	H	0.11574	0.75657	0.62796	H	0.34091	0.45275	0.66721
C	0.77517	0.57482	0.25617	H	0.06226	0.81522	0.63362	H	0.30749	0.41782	0.83683
C	0.75097	0.60951	0.31637	H	0.21369	0.96048	0.86788	H	0.3667	0.49806	0.82741

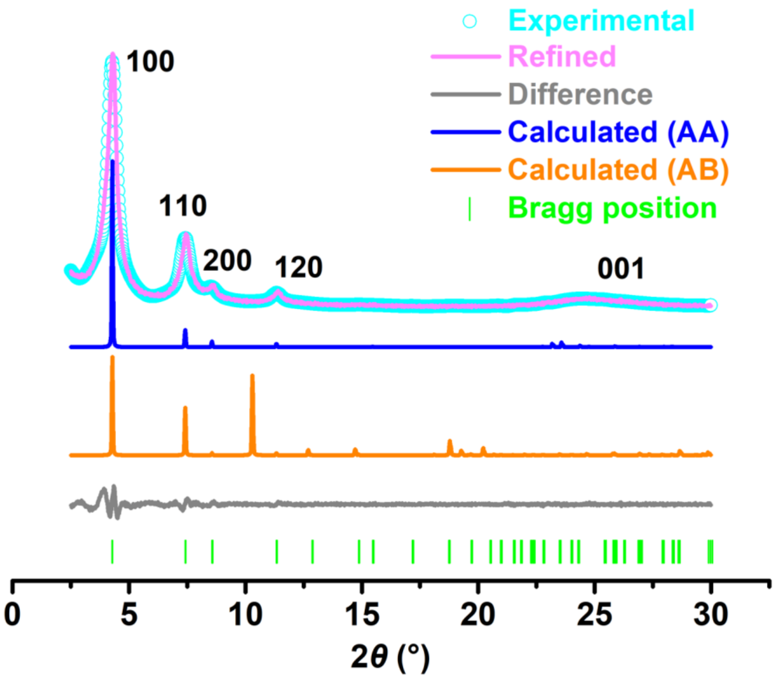

Supplementary Figure 22. JNM-100-AO refinement of the eclipsed structural model by the measured PXRD pattern using the Pawley method.

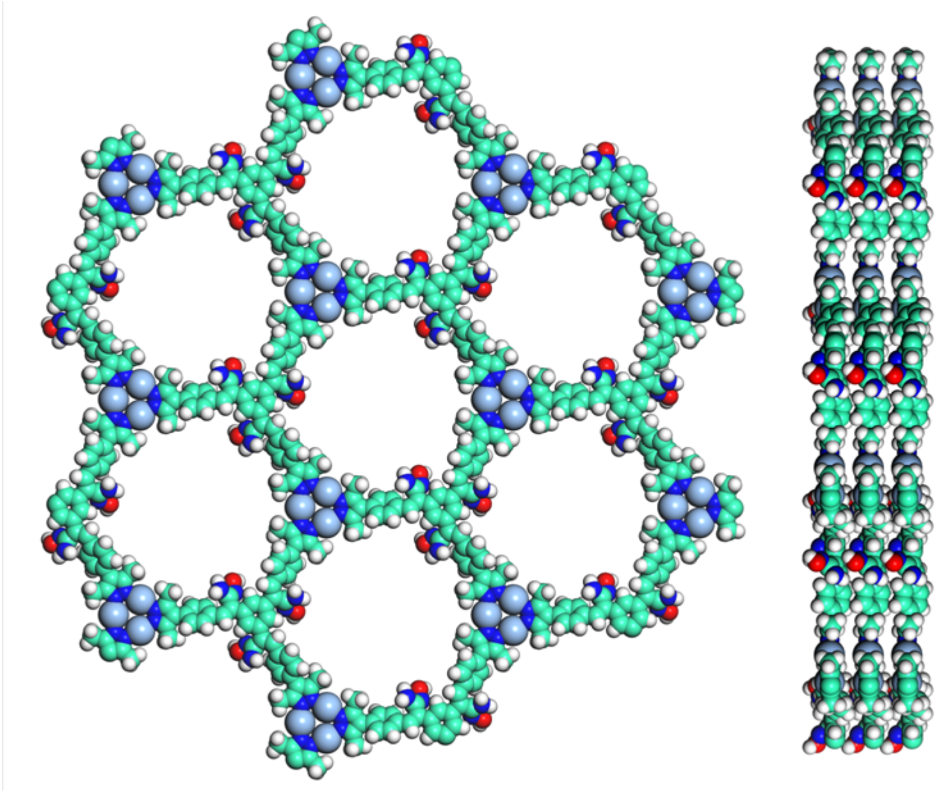

Supplementary Figure 23. Space-filling mode of JNM-100-AO in AA stacking model viewed from *c* axis(left) and *a* axis(right).

Supplementary Table 3. AA stacking model for JNM-100-AO

Hexagonal*P*-3<br>*a* = *b* = 24.9782 Å, *c* = 4.4762 Å			
Ag	0.5714	0.28703	-0.62305
N	-0.20557	0.42186	-0.61789
N	-0.20657	0.36852	-0.62174
N	0.2886	0.50594	0.14452
N	0.31302	0.45456	0.52
O	0.24218	0.45115	0.04381
C	0.37932	0.73041	-0.48589
C	0.39579	0.68409	-0.48262
C	0.42837	0.79786	-0.51378
C	-0.14654	0.46804	-0.60457
C	-0.10795	0.44281	-0.60123
C	-0.14821	0.37927	-0.61351
C	0.51613	1.0401	-0.56334
C	0.53016	1.00239	-0.73911
C	0.49727	0.93821	-0.70407
C	0.45219	0.91048	-0.47887
C	0.43708	0.94861	-0.31034
C	0.46873	1.01263	-0.35122
C	0.41584	0.84242	-0.42356
C	0.87181	0.53464	0.42674
C	0.86757	0.32892	0.3732
C	0.32292	0.50959	0.37047
H	0.44404	0.69784	-0.48715
H	0.56458	1.02225	-0.91396
H	0.5063	0.91113	-0.86153
H	0.40128	0.92893	-0.14132
H	0.45726	1.0408	-0.21095
H	0.37434	0.82824	-0.29424
H	0.898	0.56084	0.22686
H	0.90082	0.55427	0.62929
H	0.83004	0.53931	0.44826
H	0.84376	0.29854	0.17928
H	0.8518	0.30108	0.58115
H	0.91798	0.34787	0.34539
H	0.33791	0.45722	0.71195
H	0.28091	0.41066	0.44163
H	0.22335	0.4624	-0.12164

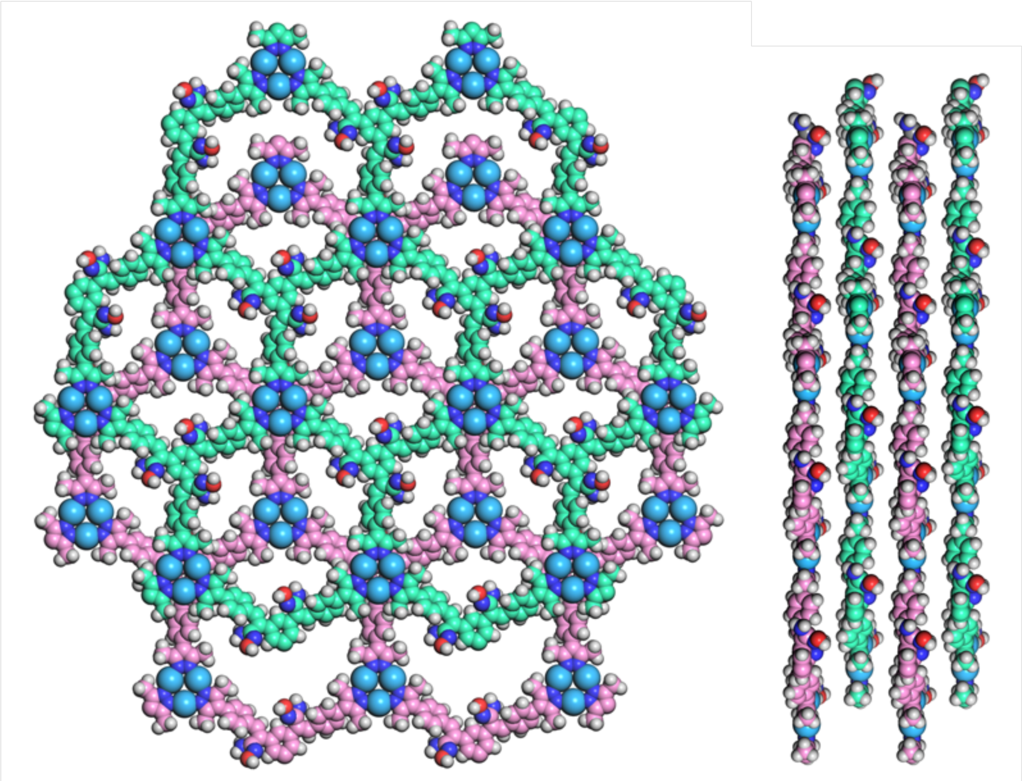

Supplementary Figure 24. Space-filling mode of JNM-100-AO in AB stacking model viewed from *c* axis(left) and *a* axis(right).

Supplementary Table4. AB stacking model for JNM-100-AO.

Hexagonal*P*-3<br>*a* = *b* = 24.9782 Å, *c* = 13.5762Å											
Ag	0.90470	0.95373	0.25278	C	0.77425	0.16943	0.22606	H	0.50506	0.2128	0.36119
Ag	0.04627	0.95107	0.25278	C	0.79159	0.21451	0.30032	H	0.77246	0.00386	0.20329
Ag	0.04893	0.09530	0.25278	C	0.84483	0.22962	0.35588	H	0.77903	0.01325	0.33598
Ag	0.23810	0.62033	0.75278	C	0.87719	0.19797	0.3424	H	0.79399	0.95743	0.27629
Ag	0.37967	0.61767	0.75278	C	0.75988	0.25086	0.31855	H	0.03476	0.21192	0.1876
Ag	0.38233	0.76190	0.75278	C	0.99613	0.79489	0.2692	H	0.03222	0.21742	0.3201
N	0.12773	0.08856	0.25448	C	0.79465	0.79913	0.25154	H	0.98543	0.23681	0.24237
N	0.12673	0.03522	0.25321	C	0.51997	0.34378	0.25064	H	0.87608	0.54738	0.36323
N	0.6219	0.17264	0.17614	C	0.04602	0.06371	0.798	H	0.92264	0.53694	0.2741
N	0.64632	0.12126	0.29994	C	0.06249	0.01739	0.79908	H	0.8709	0.42765	0.08839
N	0.91144	0.03927	0.25448	C	0.09507	0.13116	0.78881	H	0.5871	0.22266	0.29758
N	0.96478	0.09161	0.25321	C	0.52016	0.80134	0.75887	H	0.79097	0.10212	0.15686
N	0.82736	0.44937	0.17614	C	0.55875	0.77611	0.75997	H	0.73813	0.1604	0.17415
N	0.87874	0.52516	0.29994	C	0.51849	0.71257	0.75592	H	0.86095	0.26542	0.41161
N	0.96073	0.87227	0.25448	C	0.18283	0.3734	0.77246	H	0.91684	0.20944	0.38865
N	0.90839	0.87327	0.25321	C	0.19686	0.33569	0.71451	H	0.7872	0.29236	0.36119
N	0.55063	0.3781	0.17614	C	0.16397	0.27151	0.72606	H	0.99614	0.7687	0.20329
N	0.47484	0.35368	0.29994	C	0.11889	0.24378	0.80032	H	0.98675	0.76588	0.33598
N	0.46113	0.75516	0.75448	C	0.10378	0.28191	0.85588	H	0.04257	0.83666	0.27629
N	0.46013	0.70182	0.75321	C	0.13543	0.34593	0.8424	H	0.78808	0.82294	0.1876
N	0.9553	0.83924	0.67614	C	0.08254	0.17572	0.81855	H	0.78258	0.8149	0.3201
N	0.97972	0.78786	0.79994	C	0.53851	0.86794	0.7692	H	0.76319	0.74872	0.24237
N	0.24484	0.70587	0.75448	C	0.53427	0.66222	0.75154	H	0.45262	0.32879	0.36323

N	0.29818	0.75821	0.75321	C	0.98962	0.84289	0.75064	H	0.46306	0.38579	0.2741
N	0.16076	0.11597	0.67614	C	0.93629	0.98221	0.798	H	0.57235	0.44335	0.08839
N	0.29413	0.53887	0.75448	C	0.86884	0.96381	0.78881	H	0.11074	0.03114	0.79758
N	0.24179	0.53987	0.75321	C	0.19866	0.71872	0.75887	H	0.23128	0.35555	0.65686
N	0.88403	0.0447	0.67614	C	0.22389	0.78254	0.75997	H	0.173	0.24443	0.67415
N	0.80824	0.02028	0.79994	C	0.28743	0.80582	0.75592	H	0.06798	0.26223	0.91161
O	0.57548	0.11785	0.14294	C	0.6266	0.80932	0.77246	H	0.12396	0.3741	0.88865
O	0.88215	0.45773	0.14294	C	0.66431	0.86107	0.71451	H	0.04104	0.16154	0.86119
O	0.54227	0.42452	0.14294	C	0.72849	0.89235	0.72606	H	0.5647	0.89414	0.70329
O	0.90888	0.78445	0.64294	C	0.75622	0.87501	0.80032	H	0.56752	0.88757	0.83598
O	0.21555	0.12433	0.64294	C	0.71809	0.82177	0.85588	H	0.49674	0.87261	0.77629
O	0.87567	0.09112	0.64294	C	0.65407	0.78941	0.8424	H	0.51046	0.63184	0.6876
C	0.71262	0.39711	0.298	C	0.82428	0.90672	0.81855	H	0.5185	0.63438	0.8201
C	0.72909	0.35079	0.29908	C	0.13206	0.67047	0.7692	H	0.58468	0.68117	0.74237
C	0.76167	0.46456	0.28881	C	0.33778	0.87195	0.75154	H	0.00461	0.79052	0.86323
C	0.18676	0.13474	0.25887	C	0.15711	0.14663	0.75064	H	0.94761	0.74396	0.7741
C	0.22535	0.10951	0.25997	C	0.01779	0.95398	0.798	H	0.89005	0.7957	0.58839
C	0.18509	0.04597	0.25592	C	0.955	0.93751	0.79908	H	0.96886	0.0795	0.79758
C	0.84943	0.7068	0.27246	C	0.03619	0.90493	0.78881	H	0.64445	0.87563	0.65686
C	0.86346	0.66909	0.21451	C	0.28128	0.47984	0.75887	H	0.75557	0.92847	0.67415
C	0.83057	0.60491	0.22606	C	0.21746	0.44125	0.75997	H	0.73777	0.80565	0.91161
C	0.78549	0.57718	0.30032	C	0.19418	0.48151	0.75592	H	0.6259	0.74976	0.88865
C	0.77038	0.61531	0.35588	C	0.19068	0.81717	0.77246	H	0.83846	0.8794	0.86119
C	0.80203	0.67933	0.3424	C	0.13893	0.80314	0.71451	H	0.10586	0.67046	0.70329
C	0.74914	0.50912	0.31855	C	0.10765	0.83603	0.72606	H	0.11243	0.67985	0.83598
C	0.20511	0.20134	0.2692	C	0.12499	0.88111	0.80032	H	0.12739	0.62403	0.77629
C	0.20087	0.99562	0.25154	C	0.17823	0.89622	0.85588	H	0.36816	0.87852	0.6876
C	0.65622	0.17629	0.25064	C	0.21059	0.86457	0.8424	H	0.36562	0.88402	0.8201
C	0.60289	0.31561	0.298	C	0.09328	0.91746	0.81855	H	0.31883	0.90341	0.74237
C	0.64921	0.3784	0.29908	C	0.32953	0.46149	0.7692	H	0.20948	0.21398	0.86323
C	0.53544	0.29721	0.28881	C	0.12805	0.46573	0.75154	H	0.25604	0.20354	0.7741
C	0.86526	0.05212	0.25887	C	0.85337	0.01038	0.75064	H	0.2043	0.09425	0.58839
C	0.89049	0.11594	0.25997	H	0.77734	0.36454	0.29758	H	0.9205	0.88926	0.79758
C	0.95403	0.13922	0.25592	H	0.89788	0.68895	0.15686	H	0.12437	0.76872	0.65686
C	0.2932	0.14272	0.27246	H	0.8396	0.57783	0.17415	H	0.07153	0.827	0.67415
C	0.33091	0.19447	0.21451	H	0.73458	0.59563	0.41161	H	0.19435	0.93202	0.91161
C	0.39509	0.22575	0.22606	H	0.79056	0.7075	0.38865	H	0.25024	0.87604	0.88865
C	0.42282	0.20841	0.30032	H	0.70764	0.49494	0.36119	H	0.1206	0.95896	0.86119
C	0.38469	0.15517	0.35588	H	0.2313	0.22754	0.20329	H	0.32954	0.4353	0.70329
C	0.32067	0.12281	0.3424	H	0.23412	0.22097	0.33598	H	0.32015	0.43248	0.83598
C	0.49088	0.24012	0.31855	H	0.16334	0.20601	0.27629	H	0.37597	0.50326	0.77629
C	0.79866	0.00387	0.2692	H	0.17706	0.96524	0.1876	H	0.12148	0.48954	0.6876
C	0.00438	0.20535	0.25154	H	0.1851	0.96778	0.3201	H	0.11598	0.4815	0.8201
C	0.82371	0.48003	0.25064	H	0.25128	0.01457	0.24237	H	0.09659	0.41532	0.74237
C	0.68439	0.28738	0.298	H	0.67121	0.12392	0.36323	H	0.78602	0.99539	0.86323
C	0.6216	0.27091	0.29908	H	0.61421	0.07736	0.2741	H	0.79646	0.05239	0.7741
C	0.70279	0.23833	0.28881	H	0.55665	0.1291	0.08839	H	0.90575	0.10995	0.58839
C	0.94788	0.81324	0.25887	H	0.63546	0.4129	0.29758	H	0.50506	0.2128	0.36119
C	0.88406	0.77465	0.25997	H	0.31105	0.20903	0.15686	H	0.77246	0.00386	0.20329
C	0.86078	0.81491	0.25592	H	0.42217	0.26187	0.17415	H	0.77903	0.01325	0.33598
C	0.85728	0.15057	0.27246	H	0.40437	0.13905	0.41161	H	0.79399	0.95743	0.27629

12. Photoluminescent properties

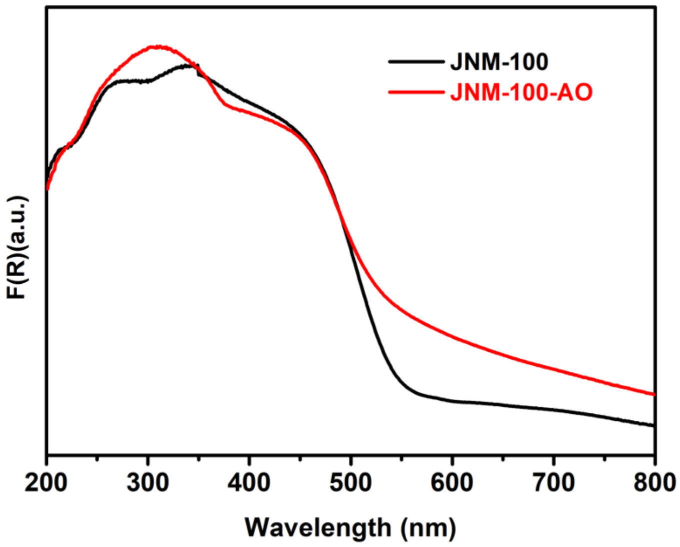

Supplementary Figure 25. Solid-state UV-Vis spectra of JNM-100 (black line) and JNM-100-AO (red line).

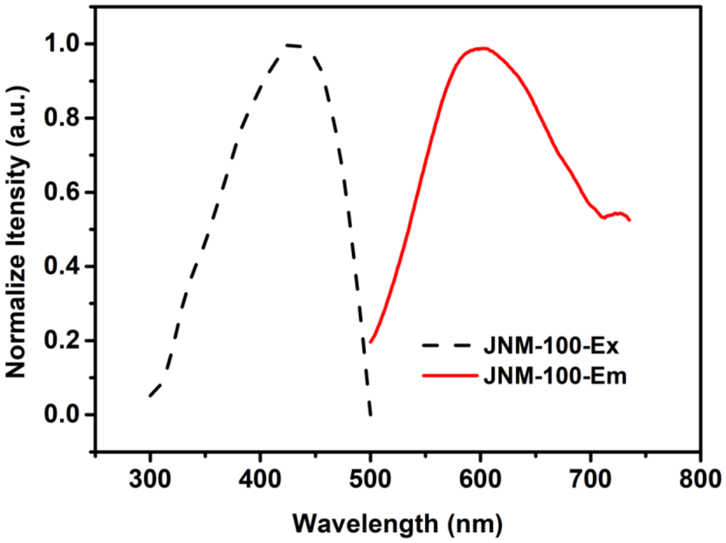

Supplementary Figure 26. Excitation (black dash line) and emission (red line) spectra of JNM-100.

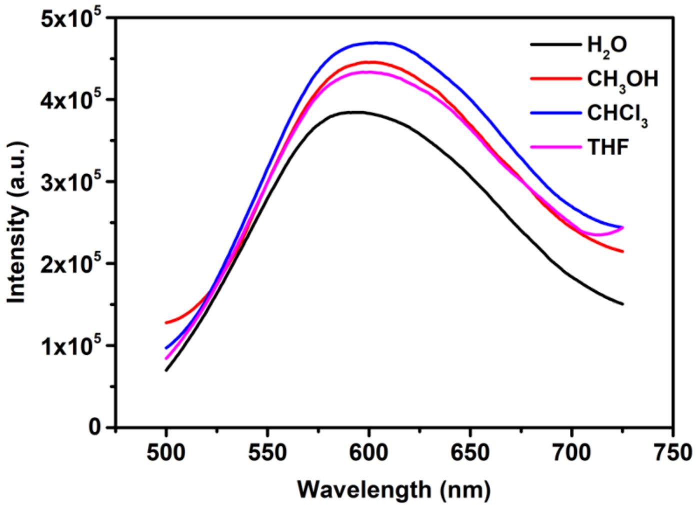

Supplementary Figure 27. Emission spectra of JNM-100 in various solvents.

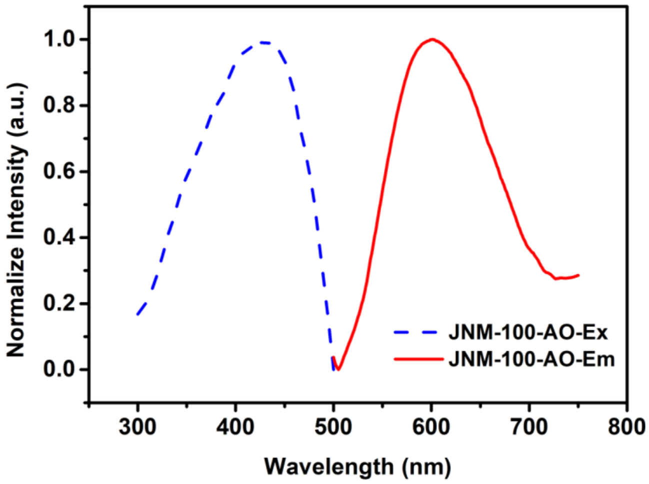

Supplementary Figure 28. Excitation (blue dash line) and emission (red line) spectra of JNM-100-AO.

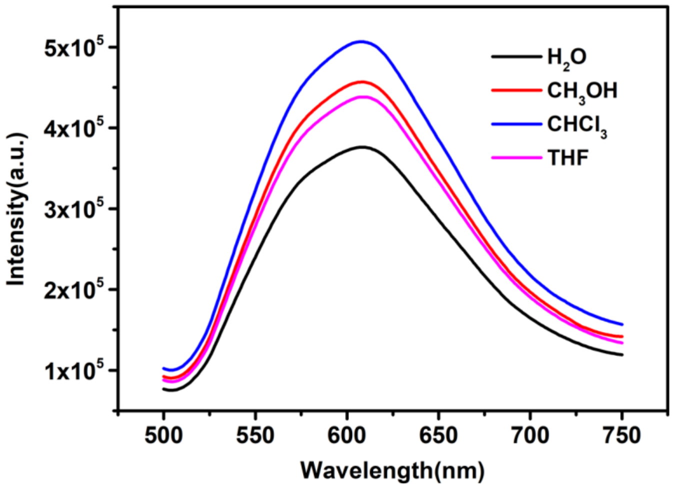

Supplementary Figure 29. Emission spectra of JNM-100-AO in various solvents.

Supplementary Table 5. Photophysical data for JNM-100 and JNM-100-AO.

Material	Excitation (nm)	Emission (nm)	QY (%)	τ_{av} (ns)
JNM-100	460	601	4.1	0.77
JNM-100-AO	460	604	3.4	0.86

13. Detection of gold

13.1 Fluorescence sensing experiments in methanol/water mixture

The stock solution of JNMs (0.3 mg/mL, 500 μL) prepared by dispersion of JNMs in water and CH_3OH mixture (50:50 v/v%) was added to the solution which contains different amounts of Au^{3+} (200 μL), where the $KAuCl_4$ was used as gold sources, diluted to 2 mL with water and methanol mixture (50:50 v/v%) in a quartz cuvette. JNMs was readily dispersed in water and methanol mixture (50:50 v/v%) and the obtained suspension was almost transparent. The fluorescence spectra were recorded immediately after an appropriate aliquot of the stock solution of ions was added. All measurements were excited at λ_{ex} = 460 nm and the corresponding emission wavelengths were tested from λ_{em} = 500 to 800 nm unless otherwise stated. After the addition of the ions stock solutions, the shape of the emission spectra did not change.

The LOD can be calculated using Supplementary Equation 1:

$$LOD = \frac{3\times S.D}{k} \qquad \text{(Supplementary Equation 1)}$$

Where k is the slope of the curve equation, and S.D. represents the standard deviation for the **JNMs** solution intensity in the absence of Au^{3+}.

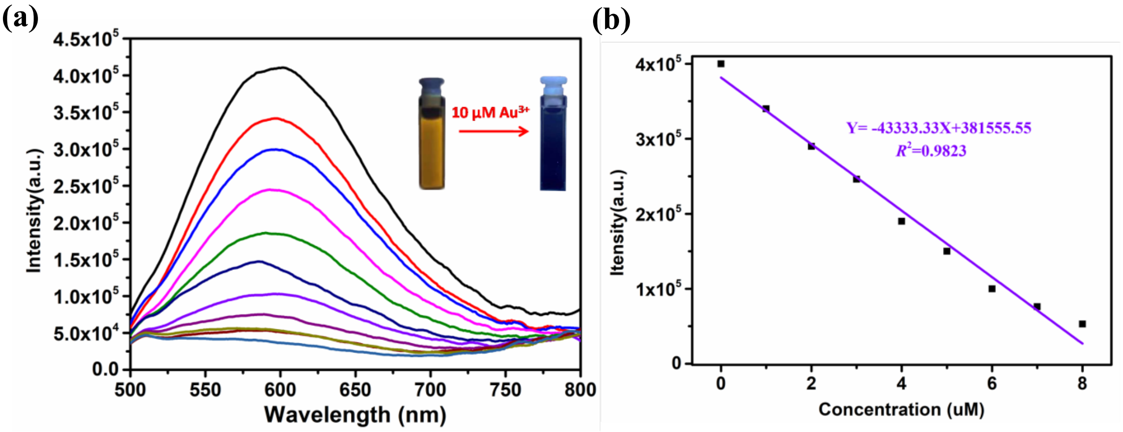

Supplementary Figure 30. Sensing experiments of gold. (a) Fluorescence spectra of JNM-100 in the presence of increasing concentration of Au(III) (from 0 to 10 μM) in water and methanol mixture (50:50 v/v%). (b) Profiles of the emission intensity *versus* Au(III) concentration showing a good linear relationship.

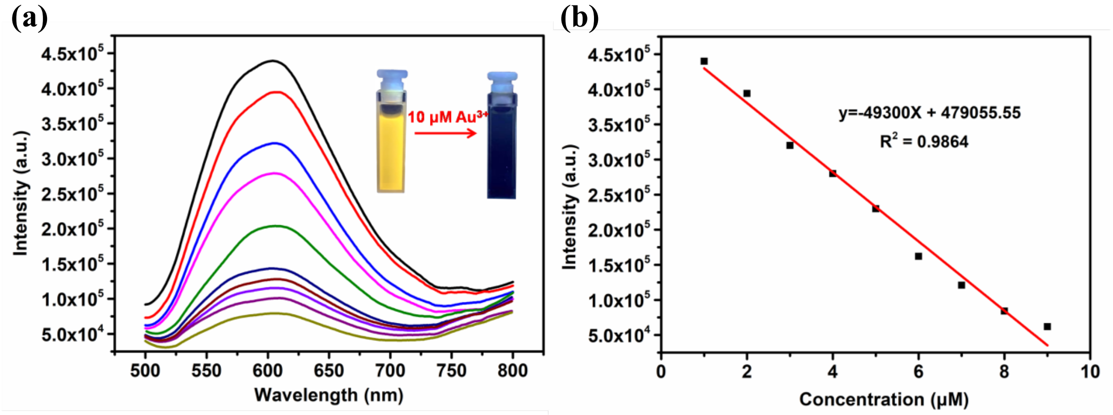

Supplementary Figure 31. Sensing experiments of gold. (a) Fluorescence spectra of JNM-100-AO in the presence of increasing concentration of Au(III) (from 0 to 10 μM) in water and methanol mixture (50:50 v/v%). (b) Profiles of the emission intensity *versus* Au(III) concentration showing a good linear relationship.

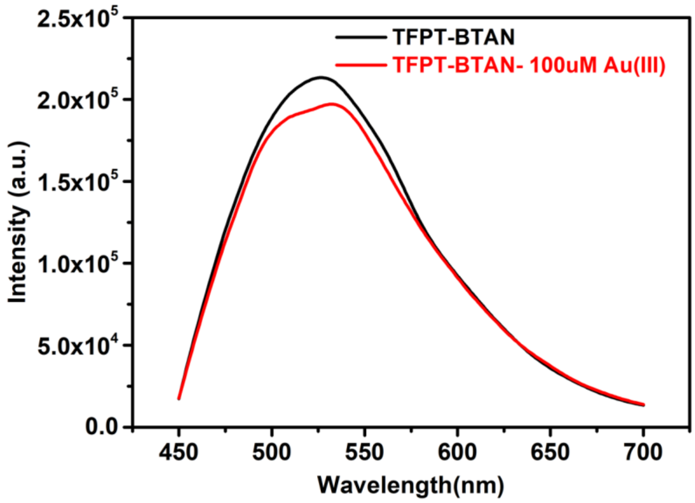

Supplementary Figure 32. Sensing experiments of gold. Fluorescence spectra of TFPT-BTAN before (black line) and after (red line) absorption of Au(III) (100 μM) in water and methanol mixture (50:50 v/v%), implying the TFPT-BTAN did not response to the Au(III) ions.

13.2 Selective detection of gold

The ions stock solutions (1mM) were prepared by dissolving the corresponding metal salts of NaCl, KCl, $CaCl_2$, $MgCl_2$, $AlCl_3$, $ZnCl_2$, $Cu(NO_3)_2\cdot 3H_2O$, $Cd(NO_3)_2\cdot 4H_2O$, $Mn(NO_3)_2$, $BaCl_2\cdot 2H_2O$, $NiCl_2\cdot 6H_2O$, $ZnCl_2$, $HgCl_2$, $Pb(NO_3)_2$, $ZrCl_4$, $Cr(NO_3)_3\cdot 9H_2O$, $FeCl_2\cdot 4H_2O$, $FeCl_3$, $Pd(NO_3)_2\cdot 2H_2O$, K_2PtCl_6, K_2PtCl_4 and $KAuCl_4$ in ultrapure water and CH_3OH mixture (50:50 v/v%). Stock solution of JNM-100, JNM-100-AO and TFPT-BTAN (0.3 mg/mL) was prepared by dispersion of JNM-100, JNM-100-AO and TFPT-BTAN in ultrapure water and CH_3OH mixture (50:50 v/v%).

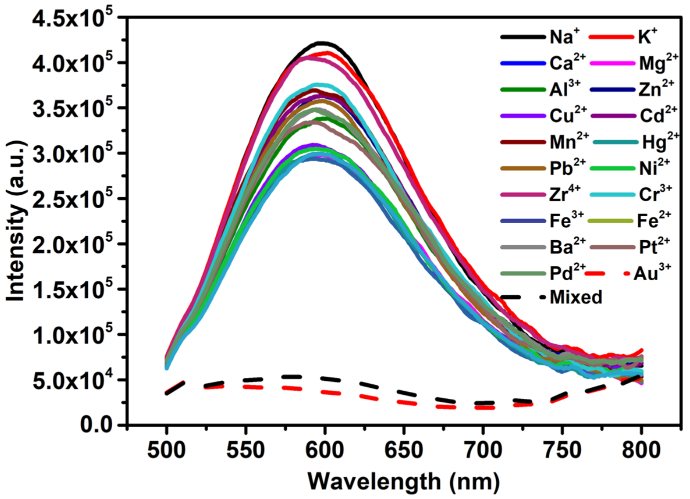

Supplementary Figure 33. The emission spectra of JNM-100 in the presence of various cations and mixed ions. Concentrations of Au^{3+} and other metal ions were 10 μM and 50 μM, respectively

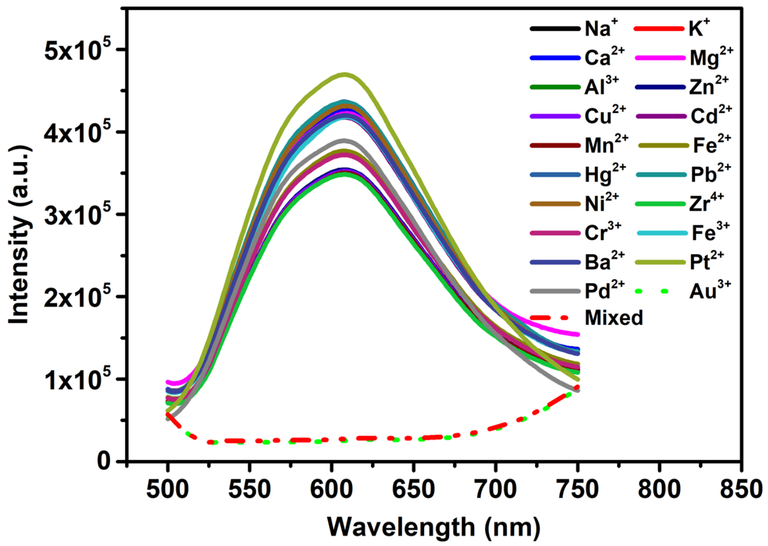

Supplementary Figure 34. The emission spectra of JNM-100-AO in the presence of various cations and mixed ions. Concentrations of Au^{3+} and other metal ions were 10 μM and 50 μM, respectively

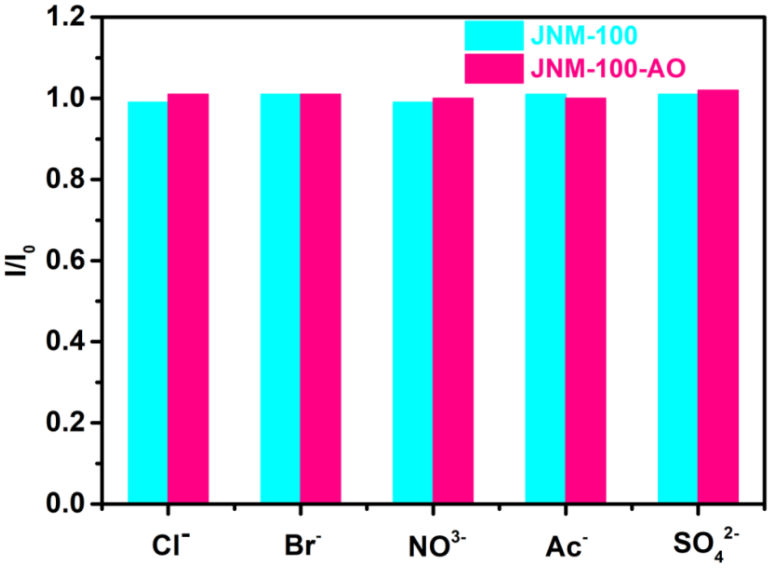

Supplementary Figure 35. Fluorescence intensity of JNM-100 and JNM-100-AO at 605 nm in the presence of various anion.

13.3 Fluorescence sensing experiments in pure water

The stock solution of JNMs (0.3 mg/mL, 500 μL) prepared by dispersion of JNMs in water was added to the solution which contains different amounts of Au^{3+} (200 μL), where the $KAuCl_4$ was used as gold sources, then diluted to 2 mL water in a quartz cuvette. JNMs was readily dispersed in water and the obtained suspension was almost transparent. The fluorescence spectra were recorded immediately after an appropriate aliquot of the stock solution of ions was added. All measurements were excited at λ_{ex} = 460 nm and the corresponding emission wavelengths were tested from λ_{em} = 500 to 800 nm unless otherwise stated.

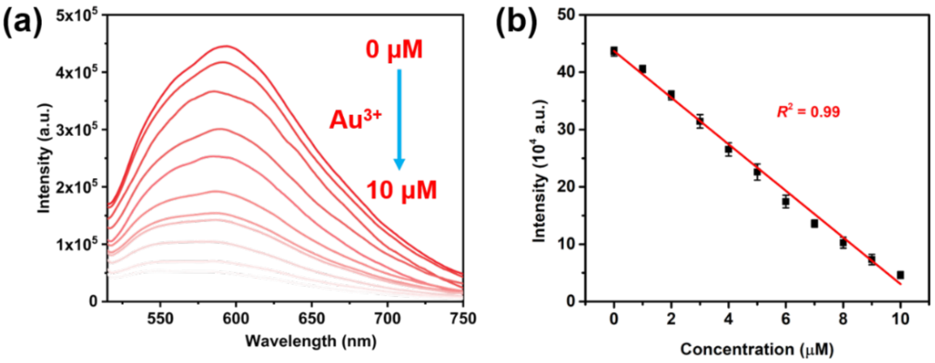

Supplementary Figure 36. Determination of LOD. (a) Fluorescence spectra of JNM-100 in the presence of increasing concentration of Au(III) (from 0 to 10 μM) in water. (b) Profiles of the emission intensity versus Au(III) concentration showing a good linear relationship, revealing LOD = 126 ppb.

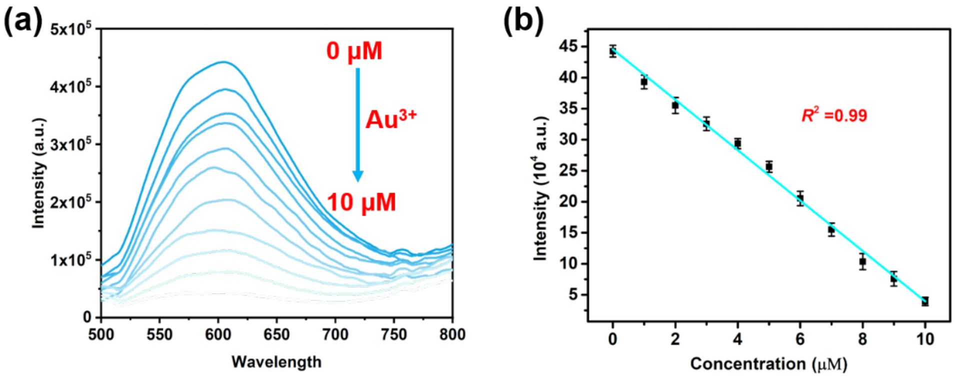

Supplementary Figure 37. Determination of LOD. (a) Fluorescence spectra of JNM-100-AO in the presence of increasing concentration of Au(III) (from 0 to 10 μM) in water. (b) Profiles of the emission intensity versus Au(III) concentration showing a good linear relationship, revealing LOD = 103 ppb.

13.4 Recovery of PL intensity of JNMs

The emission of JNM-100-AO can be quenched after addition of gold solution (same experiment procedures as 13.3), and the aqueous solution of thiourea (1.0 M, 100 μL, pH = 2 adjusted with concentrated HCl) was added to resulted mixture. After 1 hour, the emission of JNM-100-AO can be recovered and reached ~ 95% of its original intensity.

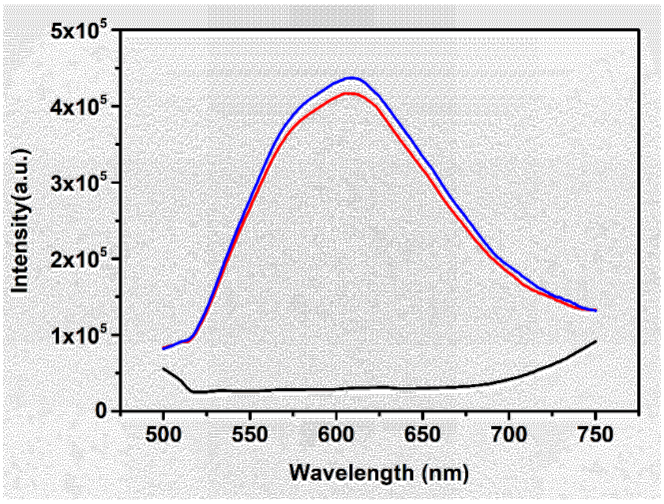

Supplementary Figure 38. Recovery of PL intensity of JNMs. Blue line: the emission spectra of JNM-100-AO in pure water (0.3 mg/mL); Black line: after addition of the aqueous solution of $KAuCl_4$ (10 μM, 200 μL), the emission was completely quenched; Red line: the aqueous solution of thiourea (1 M, 100 μL, pH = 2 adjusted with concentrated HCl) was added to above-resulted mixture, the emission of JNM-100-AO can be recovered after 1 h.

13.5 Detection of gold in industrial plating waste water

One of the important industry applications of gold is plating, and we have tried to detect the gold in the plating waste solutions considering the real application for sensing gold ions in industry. The gold plating waste solutions (pH = 6) obtained from Guangdong Guanghua Sci-Tech Co., Ltd., and it contained gold, copper, and iron with concentration of 9.5, 23.4, and 28.4 ppm. Besides of these metal ions, it also contained high concentration of CN^- anion and organic additives like HCHO. When the JNMs were added to the gold plating waste solutions, the emission was completely quenched, indicating JNMs were also promising for sensing trace amount of gold in typical industrial waste solutions.

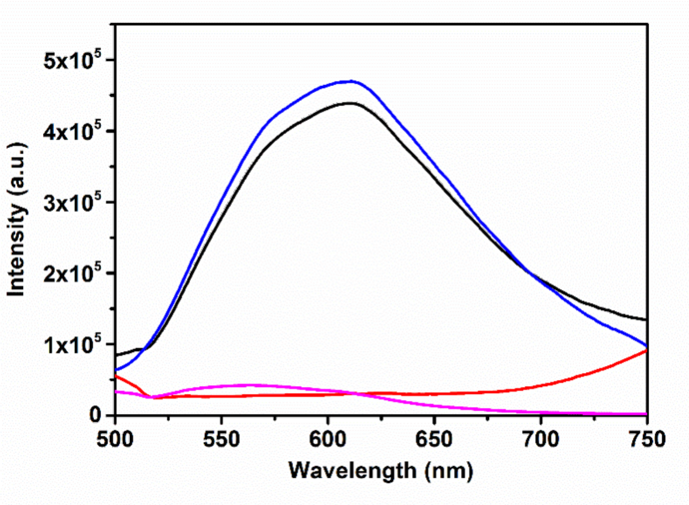

Supplementary Figure 39. Detection of gold in industrial plating waste water. The emission spectra of JNM-100 (black line) and JNM-100-AO (blue line) before and after immersing into industrial gold plating waste water (red and pink line for JNM-100 and JNM-100-AO, respectively).

14. Adsorption of gold

General procedures. In a typical adsorption experiment, 10 mg of JNMs or reference COF (TFPT-BTAN) were mixed with 10 mL of 50 mg L^{-1} Au(III) solution ($KAuCl_4$ as gold sources, pH = 2 adjusted with concentrated HCl). After reaching adsorption equilibrium, the solid was isolated *via* filtration with 0.22 μm membrane. The filtrate was analyzed using inductively coupled plasma mass spectrometry (ICP-MS) for determination of Au. The studies on the adsorption of mixed metal ions were performed in the same procedures by replacing the Au(III) solution with the solution of mixed metal ions.

Effects of Adsorption Time on the Adsorption. 10 mg JNMs was mixed with 10 mL of 100 ppm Au(III) solution (pH = 2 adjusted with concentrated HCl) for a different time (60-3600 s). The mixture was filtered, and the filtrate was collected for ICP-MS determination of Au(III).

The pseudo-first-order kinetics equation is expressed as Supplementary Equation 2:

$$\ln\,(q_{\mathrm{e}} - q_{\mathrm{t}}) = \ln q_{\mathrm{e}} - k_1 t \qquad \text{(Supplementary Equation 2)}$$

The pseudo-second-order kinetics equation is shown as Supplementary Equations 3 and 4:

$$\frac{dq_t}{dt} = k_2(q_{\mathrm{e}} - q_{\mathrm{t}})^2 \qquad \text{(Supplementary Equation 3)}$$

$$\frac{\mathrm{t}}{q_{\mathrm{t}}} = \frac{1}{k_2 q_{\mathrm{e}}^2} + \frac{\mathrm{t}}{q_{\mathrm{e}}} \qquad \text{(Supplementary Equation 4)}$$

Where q_t is the adsorption capacity (mg g^{-1}) at a predetermined time t (min) and q_e is the equilibrium adsorption capacity (mg g^{-1}). k_1 (min^{-1}) and k_2 (g mg^{-1} min^{-1}) is the rate constant of pseudo-first-order and pseudo-second-order adsorption, respectively.

The distribution coefficient (K_d) value as used for the determination of the affinity and selectivity of sorbents for Au(III) (mL g^{-1}), is given by the Supplementary Equation 5:

$$K_d = \frac{(C_0 - C_e)}{C_e} \times \frac{V}{m} \qquad \text{(Supplementary Equation 5)}$$

Where V is the volume of the treated solution (mL), m is the amount of used adsorbent (g), and C_0 and C_e are the initial concentration and the final equilibrium concentration

of Au (III) (mg L^{-1}), respectively. In the present work, 1 ppm Au(III) aqueous solutions (pH = 2 adjusted with concentrated HCl) were treated by various adsorbents overnight at a V/m ratio of 1000 mL g^{-1}.

Adsorption Isotherms and Thermodynamics. 1 mg JNM-100-Materials was mixed with 10 mL of 5-1000ppm Au(III) solution (pH = 2 adjusted with concentrated HCl) at predetermined temperature (25-55°C) till equilibrium. The mixture was filtered, and the filtrate was collected for ICP-MS determination of Au(III).

The adsorption capacity (q_t, mg g^{-1}) is calculated from the following Supplementary Equation 6.

$$q_{\mathrm{t}} = \frac{(C_0 - C_{\mathrm{t}})V}{\mathrm{m}} \qquad \text{(Supplementary Equation 6)}$$

Where C_0 (mg L^{-1}) is the initial concentrations of Au(III). C_t (mg L^{-1}) is the concentration of Au(III) at t min. V (L) is the volume of aqueous phase, m (g) is the weight of the adsorbent.

The Langmuir model equation is given as Supplementary Equation 7:

$$q_{\mathrm{e}} = \frac{C_{\mathrm{e}} q_{\mathrm{m}} K_{\mathrm{L}}}{C_{\mathrm{e}} K_{\mathrm{L}} + 1} \qquad \text{(Supplementary Equation 7)}$$

Where q_m (mg g^{-1}) is the maximum adsorption capacity, q_e (mg g^{-1}) is the equilibrium adsorption capacity, C_e (mg L^{-1}) is the equilibrium concentration of Au(III), K_{L} (L mg^{-1}) is the Langmuir constant

The thermodynamic parameters are calculated based on the following Supplementary Equations 8-10:

$$K_0 = \frac{q_e}{C_e} \qquad \text{(Supplementary Equation 8)}$$

$$\Delta \mathrm{G} = -RTlnK_0 \qquad \text{(Supplementary Equation 9)}$$

$$lnK_0 = \frac{\Delta S}{R} - \frac{\Delta H}{RT} \qquad \text{(Supplementary Equation 10)}$$

Where q_e (mg g^{-1}) is the equilibrium adsorption capacity, C_e (mg L^{-1}) is the equilibrium concentration, R is the universal gas constant (8.314 J mol^{-1} K $^{-1}$). The thermodynamic parameters that need to be calculated includes enthalpy change (ΔH, kJ mol^{-1}), free energy change (ΔG, kJ mol^{-1}), entropy change (ΔS, J mol^{-1} K^{-1}) and the thermodynamic

equilibrium constant (K_0). From above equations, K_0 can be obtained from the intercept of a plot of ln (q_e/C_e) against q_e. ΔH and ΔS were then obtained from the slope and intercept by plotting ln K_0 against 1/T.

Selective adsorption of Au^{3+}. The mixed solution (pH=2) containing Au^{3+} (1ppm), and 15 competitive metals (50 ppm) including Pt^{4+}, Pt^{2+}, Ag^{+}, Pd^{2+}, Fe^{3+}, Cu^{2+}, Zn^{2+}, Ba^{2+}, Ni^{2+}, Co^{2+}, Zr^{4+}, Mn^{2+}, Al^{3+}, Mg^{2+}, and Ca^{2+}, was prepared. Adding 10 mg of JNMs into 10 mL above as-prepared solution, and soaked for 1h. After then the mixture was filtrated with 0.22 μm membrane, and the filtrate was analyzed by ICP-MS.

Effect of pH on Adsorption. 10 mg JNM-100 or JNM-100-AO was mixed with 10 mL of 1000 mg L^{-1} Au(III) solution in different pH value solution (1-10) for 1h. The mixture was filtered, and the filtrate was collected for ICP-MS determination of Au.

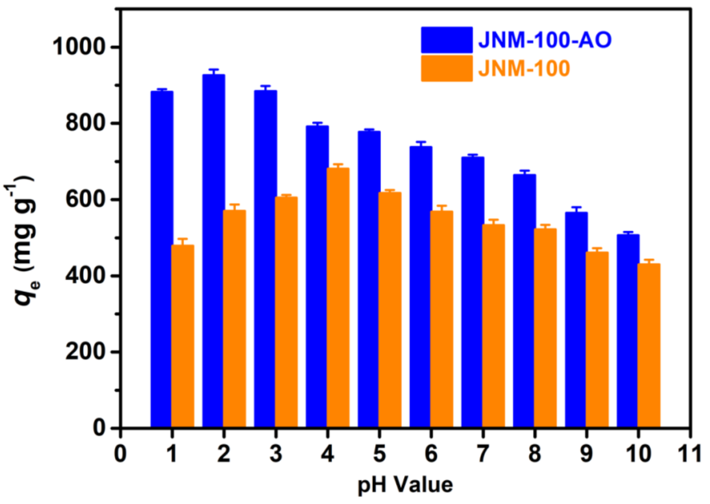

Supplementary Figure 40. Effect of pH value on the adsorption of gold on JNM-100 (orange) and JNM-100-AO (blue) at initial concentration of 1000 ppm. The error bar represents the standard deviation of three independent measurements.

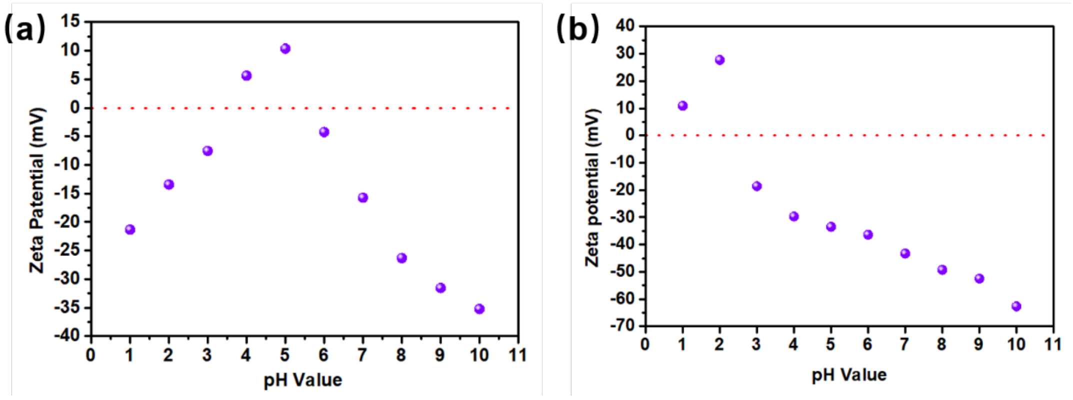

Supplementary Figure 41. The Zeta potential analysis. (a) JNM-100, and (b) JNM-100-AO under different pH values.

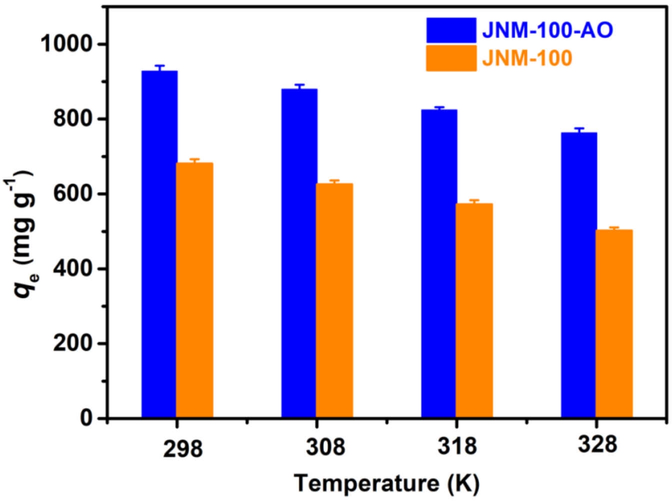

Supplementary Figure 42. Effect of temperature on the adsorption of gold on JNM-100 (orange) and JNM-100-AO (blue) at initial concentration of 1000 ppm and pH=2. The error bar represents the standard deviation of three independent measurements.

Recyclability test: The 10 mg of JNMs were added into 10 mL gold solution (C_0 = 100 or 1000 ppm, the amount of gold is 1 mg or 10 mg). After one run of adsorption, the used JNMs (10 mg) was regenerated by treatment with 30 mL thiourea (1 M, pH = 2 adjusted with HCl) solution and shaken for 5h, the resulting suspension was filtered and washed with ultra-pure water. The residue was washed with ethanol and dried under vacuum at 80 °C for 6 h to give JNMs. the resultant material was used for another adsorption experiment. It was found that after five consecutive cycles JNMs still

exhibited good adsorption ability. As shown in Supplementary Figure 43, JNMs delivered similar performances and the adsorption capacity and desorption capacity both decline with cycles increased. More importantly, in the first cycle, the JNM-100 and JNM-100-AO can adsorb 6.81 and 9.55 mg gold, but only desorb 6.75 and 9.45 mg gold, respectively, indicating the incomplete desorption with thiourea.

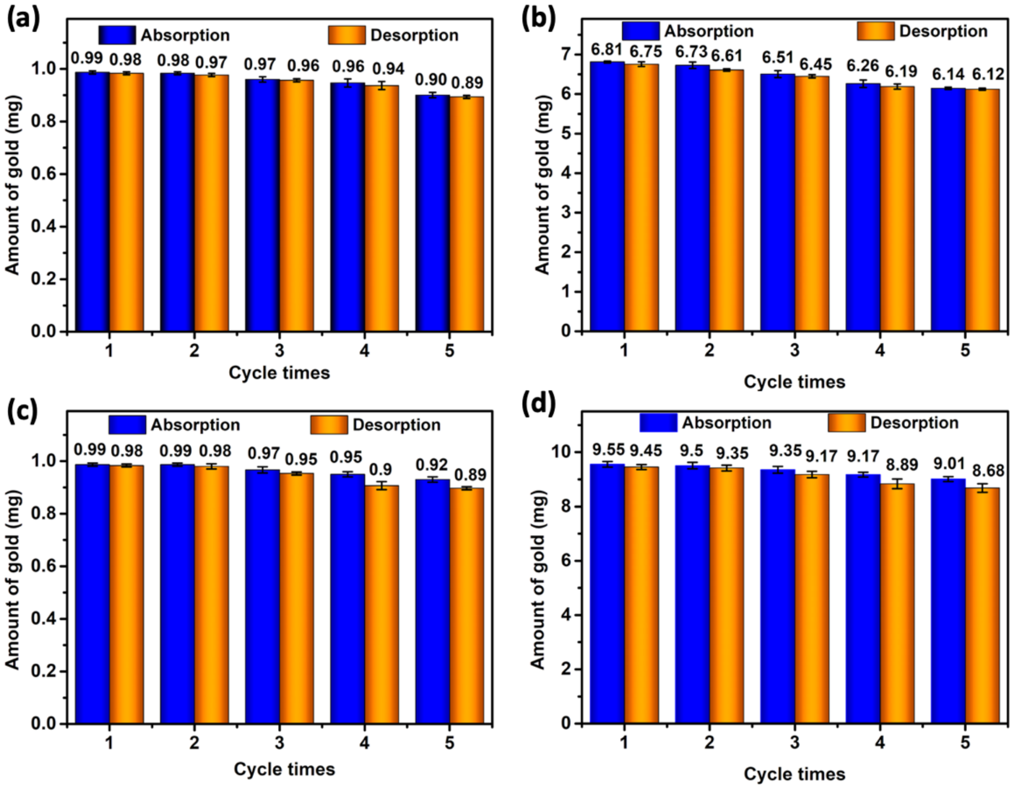

Supplementary Figure 43. Recyclability test. JNM-100 at gold concentration of 100 ppm (a), and 1000 ppm (b); and recyclability test of JNM-100-AO at gold concentration of 100 ppm (c) and 1000 ppm (d) (Blue: adsorbed gold; Orange: desorbed gold). The error bar represents the standard deviation of three independent measurements.

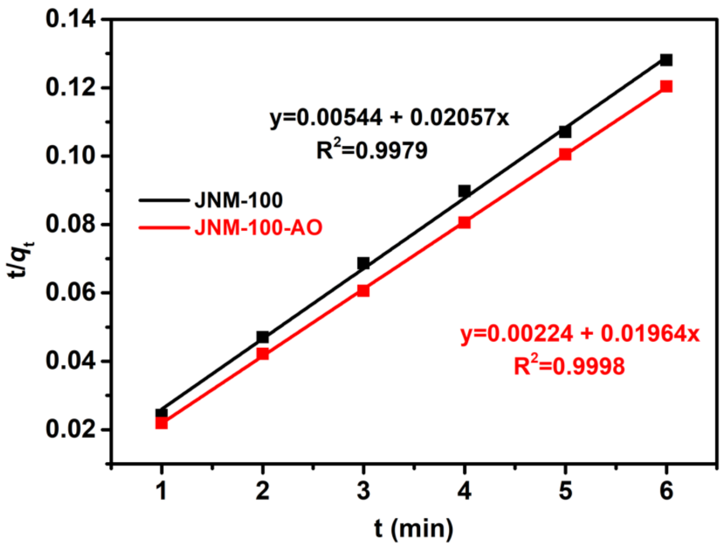

Supplementary Figure 44. The pseudo-second-order kinetic plot for the adsorption Au^{3+} ions on JNM-100 and JNM-100-AO. The adsorption kinetic process fit well with the pseudo second-order kinetic model as indicated by the high values of the correlation coefficients (R^2 = 0.9998).

Gold extraction under low concentration.

Experimental procedures: With 50 L water containing 1 ppm gold (pH = 2 adjusted with concentrated HCl), 10 mg of JNM-100-AO was put into a medium pressure tube, and the solution was circulated by water pump for 36 h (Supplementary Figure 45a). After then, ICP-MS was used to evaluate the concentration of gold in the remaining solution. In addition, the adsorbed gold materials were collected and immersed into a thiourea solution (1 M, 25 mL, pH = 2 adjusted with concentrated HCl) to extract the gold. After then, the concentration of gold in the desorbed solution was analyzed with ICP-MS. The JNM-100-AO also deliver maximum adsorption capacity of 767 mg g^{-1} under very low gold concentration of 1 ppm, indicating the high affinity of JNM-100-AO toward gold.

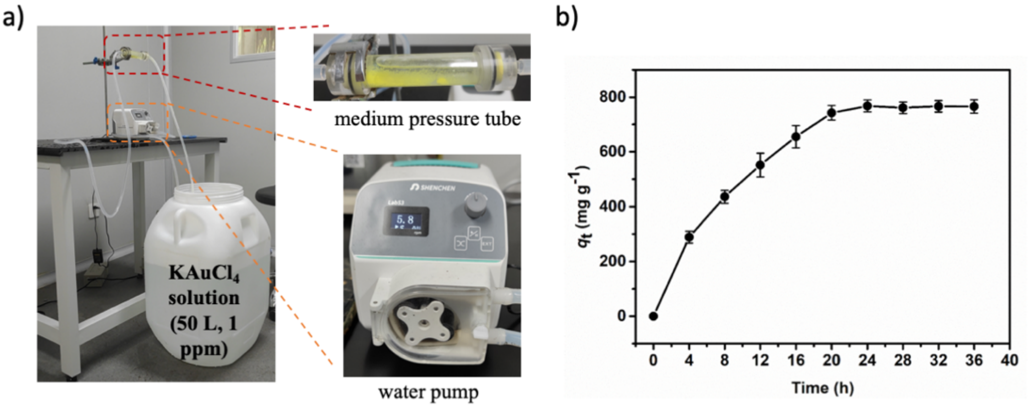

Supplementary Figure 45. The experimental set up of gold extraction under low concentration of gold (~ 1ppm). (a) The photo of experimental set up; (b) The adsorption isotherms of gold at the concentration of 1 ppm at rt using JNM-100-AO. The error bar represents the standard deviation of three independent measurements.

Supplementary Table 6. Thermodynamic parameters for the absorption of Au^{3+} on JNMs

**Absorbents**	**T (K)**	**q_e (mg/g)**	**K_0**	**ΔG(kJ/mol)**	**ΔH (kJ/mol)**	**ΔS (J/mol)**
JNM-100	298	681.93	1.36	-0.76182	-6.436007	-19.04
	308	626.23	1.57			
	318	572.55	1.43			
	328	502.44	1.26			
JNM-100-AO	298	920.51	1.54	-1.06977	-3.549906	-8.32
	308	880.24	1.76			
	318	824.40	1.64			
	328	763.19	1.52			

Supplementary Table7. Parameters of Langmuir models for the absorption of Au^{3+} on JNMs

Isotherm models	**Langmuir isotherm**		
Parameters	q_m	K_L	R^2
JNM-100	708.24	0.093	0.999
JNM-100-AO	954.31	0.095	0.998
TFPT-BTAN	167.23	0.096	0.999

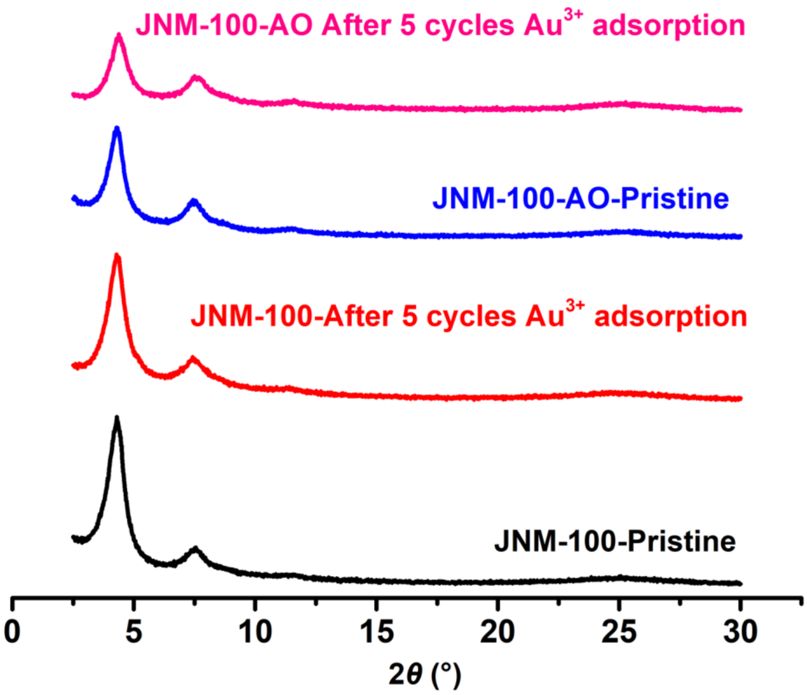

Supplementary Figure 46. PXRD for JNMs after 5 cycles Au^{3+} adsorption.

15. Extraction of gold from e-waste

NOTE 1!: The purchased CPUs have four different brands at least.

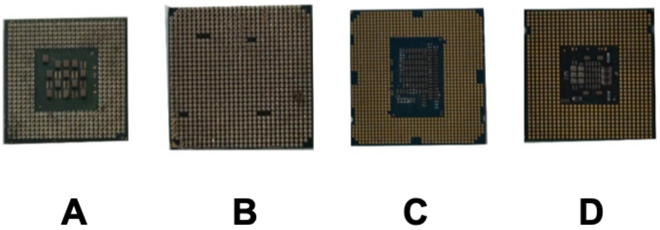

Supplementary Figure 47. The picture of purchased CPUs processors showing at least four different brands.

NOTE 2!: The amount of NBS and pyridine will affect the gold concentration in leaching solution. Specifically, with increasing amount of NBS and pyridine, the gold concentration will increase (Supplementary Table8), and for CPU A, it will reach the maximum of gold concentration of ~ 46 ppm (Supplementary Table 8).

Supplementary Table 8. The gold concentration in leaching solution with different amount of NBS and pyridine.

NBS (mg)	Pyridine (μL)	Au^{3+} Concentration (ppm)[a]
120	10	4.8
150	10	14.2
180	10	25.5
210	10	37.0
120	20	9.6
120	20	18.6
120	40	24.4
120	50	31.2
120	60	30.5
**150**	**50**	**45.3**
200	50	44.2

[a]One CUP A in Supplementary Figure 47 is used for optimizing amount of NBS and pyridine, and the volume of leaching solution is 100 mL.

NOTE 3!: The different brand of CPUs will give different gold concentration in leaching solution as shown in Supplementary Table 9. Moreover, we also tried aqua regia system for comparison, which also gave similar results with variation of gold concentration in leaching solution. Combined these results, to obtain the maximum of gold, the usage of NBS/Py should be excessive (at least more than 150 mg/50 μL per one CPU).

Supplementary Table 9. The gold concentration in leaching solution with NBS/pyridine or aqua regia.

Leaching solution	Au^{3+} Concentration (ppm)[a]			
	CPU A	CPU B	CPU C	CPU D
NBS (150 mg) / Pyrinde (50 μL)	46.2	69.2	59.2	52.1
aqua regia (5 mL)	48.7	71.9	61.4	53.5

[a]One CUP A, B, C or D in Supplementary Figure 47 is used for comparison, and the volume of leaching solution is 100 mL.

Gold recovery from electronic waste treated with NBS/Py

Although the e-wastes are commonly pretreated using aqua regia as leaching solution in industry, considering the stability of JNMs in aqua regia and environmental issues, the N-bromosuccinimide (NBS) and pyridine (Py) are used for preparing the leaching solution. It is worthy to mention that the JNMs exhibited lower affinity toward gold in aqua regia system than that in NBS/Py (See Table 10) due to high concentration of counterions such as Cl^- and NO_3^- would be competitive with $[AuCl_4]^-$. The leaching solution was prepared by mixing 100 mL of distilled water with 20 μL of pyridine (5M) and NBS (360 mg, 18 mM) (See SI for more details). The 3 CPU processors were treated with leaching solution at rt for 24 h, and the mixture was acidified to pH= 2 with concentrated HCl (Au^{3+} concentration = ~ 105 ppm analyzed by ICP-MS). After then above prepared leaching solution (8 mL) was diluted with water (92 mL), the mixture was subsequently analyzed by ICP-MS to determine the metal concentrations (Au^{3+} 8.5 ppm, Ni^{2+} 1468.2 ppm and Cu^{2+} 1865.8 ppm). After that, 10 mg of JNMs were added to 50 mL above prepared leaching solution at rt for 1h, then the mixture was filtrated with 0.22 μm membrane. The filtrate was analyzed with ICP-MS for determination of

elemental concentration.

Practical application of gold recovery from e-waste treated with aqua regia.

150 CPUs were soaked in 500 mL of aqua regia for 24 h. The leaching solution was obtained by filtration and diluted with water to a volume of 1000 mL. After then, the resulted solution was adjusted pH to 2 with NaOH, then 250 mg of JNM-100-AO added and stirred for 1 hours, and finally filter to obtain a gray brown powder JNM-100-AO-Au. The resulted powder was soaking in 100 mL 1M thiourea solution (pH=2, adjusted with HCl) and stirred 5 hours to give yellow solution and JNM-100-AO. After filtration, the JNM-100-AO was added again into leaching solution to collect residual gold, then desorbed with thiourea solution. Such processes were repeated three times. The resulted yellow solution was reduced with $Na_2S_2O_5$ to give a black powder of gold. The powder was washed three times with 50 mL water and then air-dried. Then, 10 mg of borax was mixed into the powder as a stabilizer and the mixture was sintered at ultra-high temperature until the black powder disappeared to obtain a molten golden yellow solid. The obtained gold was weighed, and 1 mg of gold was scraped and dissolved in 10 mL of aqua regia, and the purity of gold was determined by ICP-MS.

Gold recovery performance comparison.

Gold recovery performance was evaluated by gold adsorption efficiency, where $A_e = (C_0 - C)/C_0$ (C_0 represents the original gold concentration before adsorption, C represents the remain gold concentration in leaching solution after adsorption). As shown in Supplementary Table10, gold removal efficiency is estimated to be 80% after three adsorption-desorption cycles, which can be improved by further recycles. It is worthy to mention that the A_e is much higher for NBS/Pyridine system than that for aqua regia. Since the JNMs will be positive charged under acidic condition, the high concentration of counterions such as Cl^- and NO_3^- would be competitive with $[AuCl_4]^-$, leading to the lower gold removal efficiency.

Supplementary Table 10. The gold removal efficiency using NBS/pyridine or aqua regia.

Leaching solution[a]	C_0 (ppm)[b]	C (ppm)[c]	Removal efficiency (A_e)	Weight of Gold
NBS /Pyridine	755.9	151.2	80%	0.61 g
aqua regia	764.9	260.1	66%	0.50 g

[a]150 CPU containing mixed bands were used. [b]C_0 represents the original gold concentration before adsorption. The experiments were repeated three times to give the average value. [c]C represents the remain gold concentration in leaching solution after adsorption. The experiments were repeated three times to give the average value.

Selectivity calculation. The distribution coefficient (k_d) value as used for the determination of the affinity and selectivity of sorbents for Au(III) (mL g^{-1}), is given by the Supplementary Equation 11:

$$k_d = \frac{(C_0 - C_e)}{C_e} \times \frac{V}{m} \qquad \text{(Supplementary Equation 11)}$$

Where V is the volume of the treated solution (mL), m is the amount of used adsorbent (g), and C_0 and C_e are the initial concentration and the final equilibrium concentration of Au (III) (mg L^{-1}), respectively. Herein, 10 mg of JNMs was added to 50 mL leaching solution, thus V/m = 5000 mL g^{-1}.

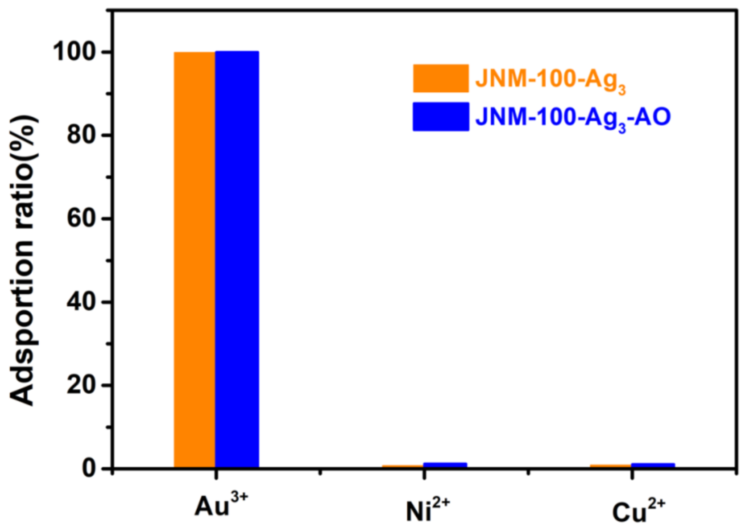

Supplementary Figure 48. Au (III) adsorption ratio of **JNM-100** (orange) and JNM-100-AO (blue) from CPU waste liquid. Initial concentration: Au^{3+} 8.5 ppm, Ni^{2+} 1468.2 ppm and Cu^{2+} 1865.8 ppm.

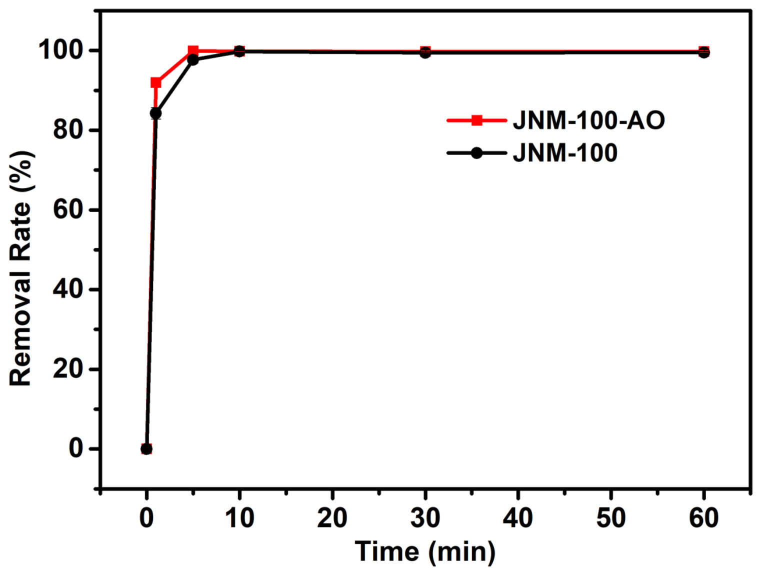

Supplementary Figure 49. Time-dependent Au (III) adsorption ratio of JNM-100 (black) and JNM-100-AO (red) from CPU waste liquid.

16. XPS spectra

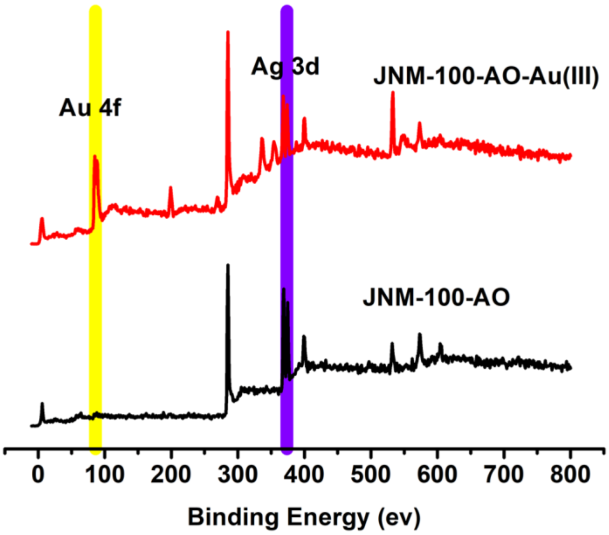

Supplementary Figure 50. The wide XPS spectra of JNM-100-AO and JNM-100-AO-Au(III), the spectra clearly showed the Au 4f peaks, supporting the gold was indeed adsorbed.

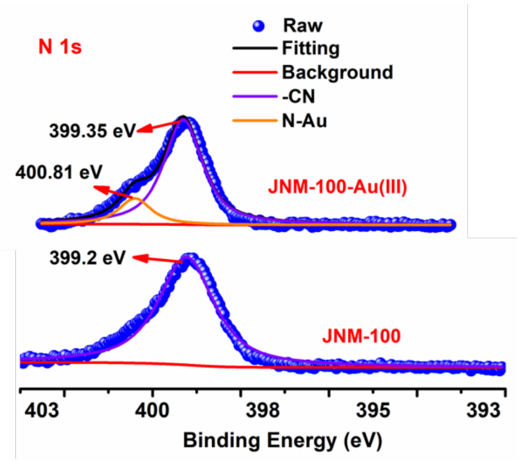

Supplementary Figure 51. The N1s XPS spectra of JNM-100 and JNM-100-Au(III).

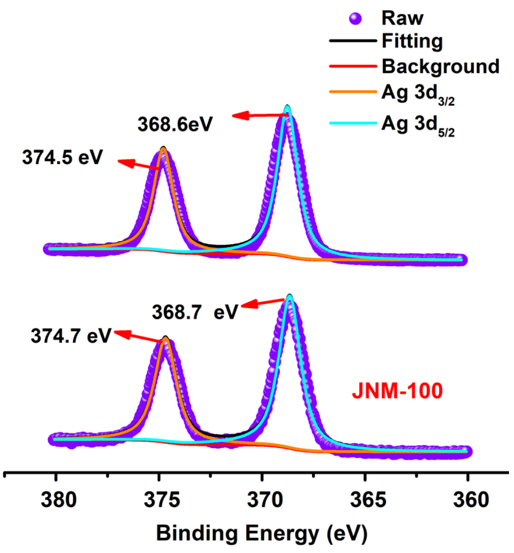

Supplementary Figure 52. The Ag 3d XPS spectra of JNM-100 and JNM-100-Au(III).

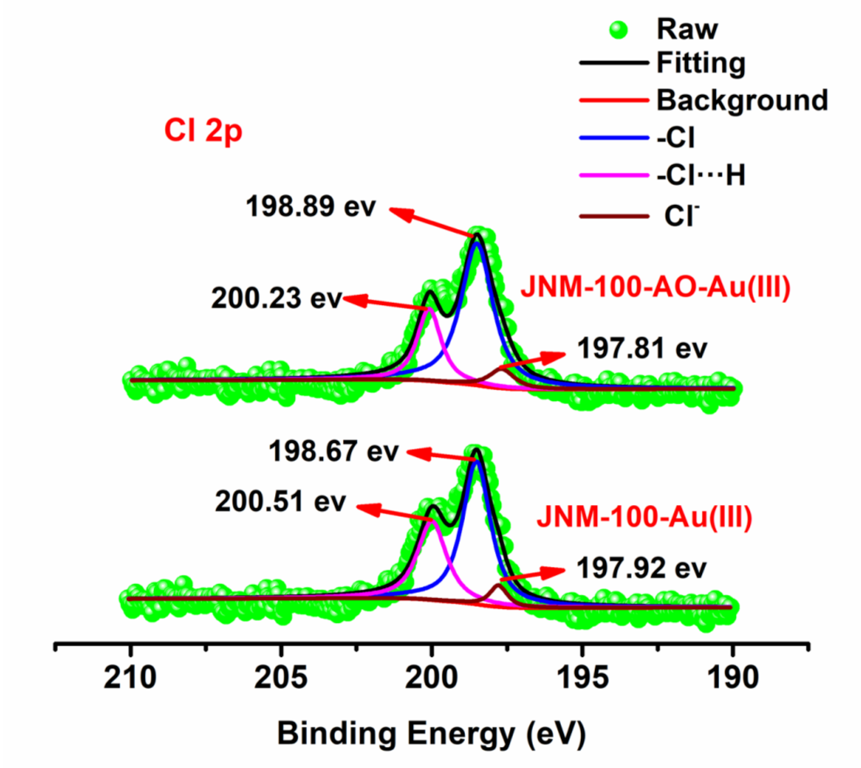

Supplementary Figure 53. The Cl 2p XPS spectra of JNM-100-Au(III) and JNM-100-AO-Au(III) .

17. DFT calculations

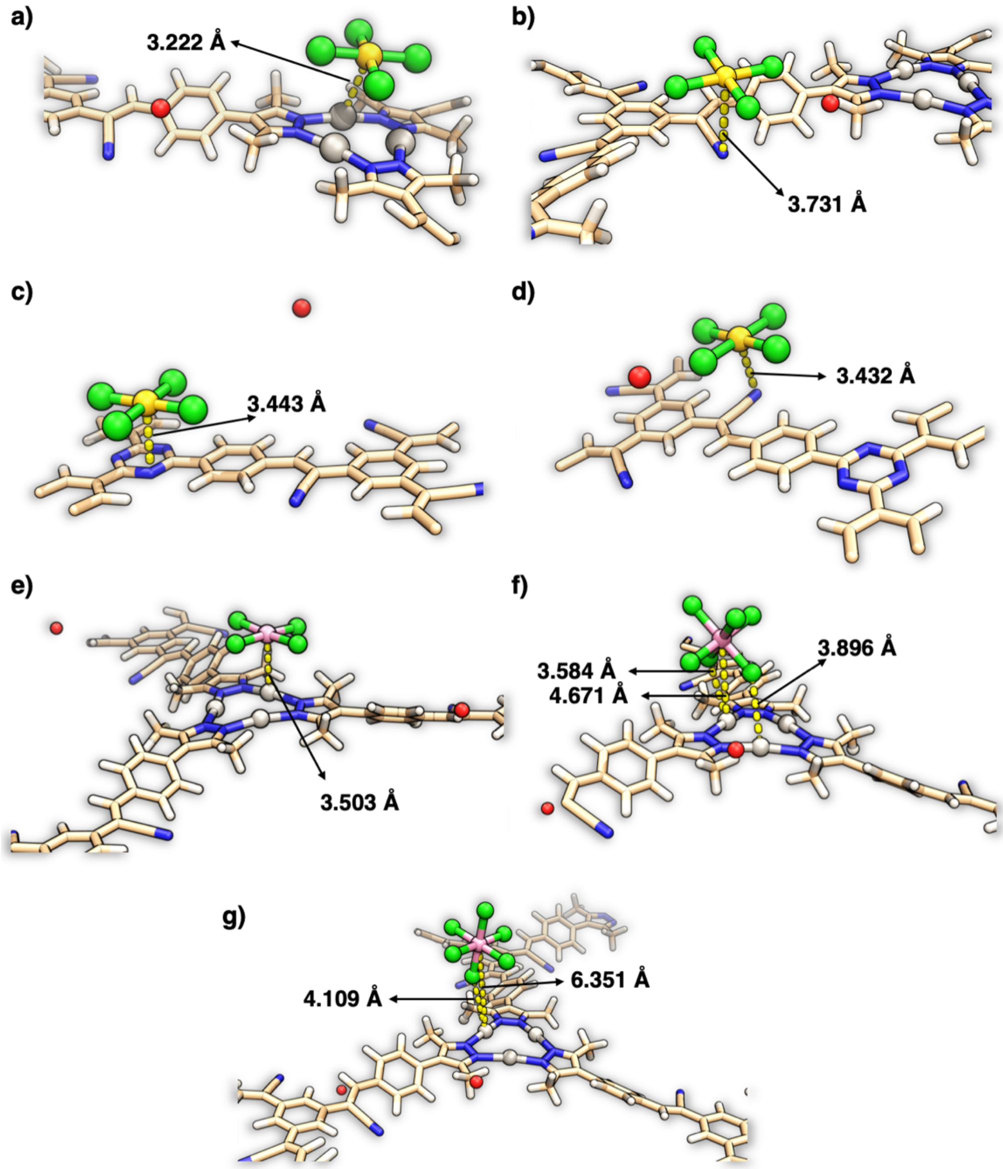

Supplementary Figure 54. Calculated periodic structure showing the interactions of different binding sites with $[AuCl_4]^-$, $[PtCl_4]^{2-}$ and $[PtCl_6]^{2-}$. $[AuCl_4]^-$ interacted with (a) Ag(I)-CTU, and (b) CN groups on JNM-100. $[AuCl_4]^-$ interacted with (c) triazine ring, and (d) CN groups on reference sp^2 COF TFPT-BTAN. (e) $[PtCl_4]^{2-}$ interacted with Ag(I)-CTU on JNM-100. (f, and g) Two possible structure of $[PtCl_6]^{2-}$ interacted with Ag(I)-CTU on JNM-100. ($[AuCl_4]^-$, $[PtCl_4]^{2-}$ and $[PtCl_6]^{2-}$ displayed in ball-stick model, and only repeat structural unit was shown for clarity. Au, yellow; Pt, pink; Cl, green; Ag, silver; H, white; C, wheat; N, blue; H^+, red.)

Supplementary Table 11. Calculated results for the JNM-100 and TFPT-BTAN for possible interactions between three active sites, $[AuCl_4]^-$, $[PtCl_4]^{2-}$ and $[PtCl_6]^{2-}$ by DFT.

	Active site	Distance [Å] Ag(N)…Au	Indicative interaction	E_b(eV)
JNM-100	Ag-CTU	3.222	metallophilic attractions	−0.478
JNM-100	−C≡N	3.731	coordinate interaction	−0.092
TFPT-BTAN	−C≡N	3.432	coordinate interaction	0.237
TFPT-BTAN	Triazine ring	3.443	coordinate interaction	0.187
		JNM-100-$[PtCl_4]^{2-}$		
JNM-100	Ag-CTU	Distance Pt….Au [Å] 3.503	metallophilic attractions	−0.268
		JNM-100-$[PtCl_6]^{2-}$		
JNM-100	Ag-CTU	Distance Ag….Cl [Å] 3.584 and 3.896	metal-halogen attractions	−0.067
JNM-100	Ag-CTU	Distance Ag….Cl [Å] 4.109	metal-halogen attractions	0.192

Supplementary Table 12. Comparison of Au^{3+} sensing and capacity of various adsorbents.

Material	Au^{3+} Capacity (mg g^{-1})	Equilibrium time(min)	System/ Trace gold concentration/ Equilibrium time(min)	Removal	Regeneration	Selectivity	Ref
JNM-100	708.24	5	Water/ 5 ppm/ 1 min	99%	√	√	This work
JNM-100-AO	954.31	3	Seawater/ 20 ppb/ 10 min	99%	√	√	
DTDD-MOF	1119	240	Water / 200 ppm/ 240 min	99%	√	√	[2]
Fe-BTC/PpPDA	934	2	Sea water/ 1 ppm / 30 min	99%	√	√	[3]
UIO-66	56	5	Water/ 100 ppm/ 45 min	90%	**N.R**	**N.R**	[4]
UIO-66-NH_2	256	30	Water/ 100 ppm / 45 min	99%.	**N.R**	**N.R**	
UIO-66-TU	326	90	Water/ 150ppm / 90 min	N.R.	√	√	[5]
UIO-66-BTU	680	240	Water/ 200 ppm/ 240 min	93%	√	√	[6]
UIO-66-TA	372	240	Water/ 900 ppm/ 240 min	N.R.	√	√	[7]
Methionine-Decorated MOF	598	30	Water:CH_3OH(1:1)/ 30 ppm/ 60 min	98%	√	√	[8]

N.R. = Not reported.

Supplementary Reference

(1) W. R. Cui, C. R. Zhang, W. Jiang, F. F. Li, R. P. Liang, J. Liu, J. D. Qiu, Regenerable and sp^2 carbon-conjugated covalent organic frameworks for selective detection and extraction of uranium. *Nat. Commun.* **2020**, *11*, 436.

(2) Z. Huang, M. Z, C. Wang, S. Wang, L. Dai, L. Zhang and Lei Xu., Selective removal mechanism of the novel Zr-based metal organic framework adsorbents for gold ions from aqueous solutions. *Chem. Eng. J.* **2020**, 384, 123343.

(3) Sun, D. T.; Yang, N. G. S.; Oveisi, E.; Queen,W. L. Rapid, selective extraction of trace amounts of gold from complex water mixtures with a metal–organic framework (MOF)/polymer composite. *J. Am. Chem. Soc.* **2018**, *140*, 16697-16703.

(4) Lin, R. S.; Reddy, D. H. K.; Bediako, J. K.; Song, M. H.; Wei, W.; Kim, J. A.; Yun, Y. S. Effective adsorption of Pd(ii), Pt(iv) and Au(iii) by Zr(iv)-based metal–organic frameworks from strongly acidic solutions. *J. Mater. Chem. A.* **2017**, *5*, 13557-13564.

(5) Wu, C.; Zhu, X.; Wang, Z.; Yang, J.; Li, Y.-S.; Gu, J.-L. Specific recovery and in situ reduction of precious metals from waste to create MOF composites with immobilized nanoclusters. *Ind. Eng. Chem. Res.* **2017**, *56*, 13975-13982.

(6) J.-K. Guo, X.-H. F, J.-Y. Wang, S.-H. Yu, M.-W. Laipan, X.-H. Ren, C. Zhang, L. Zhang, Y.-T. Li. Highly efficient and selective recovery of Au(III) from aqueous solution by bisthiourea immobilized UiO-66-NH2: Performance and mechanisms. *Chem. Eng. J.* **2021,** 425, 130588.

(7) C. Wang, G. L. J.-L. Zhao, S.-X. Wang, L.-B. Zhang, X.-T. Li, Y. Ying, Y., Highly selective recovery of Au(III) from wastewater by thioctic acid modified Zr-MOF: Experiment and DFT calculation. *Chem. Eng. J.* **2020**, 380, 122511.

(8) Mon, M.; Ferrando-Soria, J.; Grancha, T.; Fortea-Pérez, F. R.; Gascon, J.; Leyva-Pérez, A.; Armentano, D.; Pardo, E. Selective gold recovery and catalysis in a highly flexible methionine-decorated metal–organic framework. *J. Am. Chem. Soc.* **2016**, *138*, 7864-7867.
